# Molecular basis of autoimmune disease protection by MDA5 variants

**DOI:** 10.1016/j.celrep.2025.115754

**Published:** 2025-05-30

**Authors:** Rahul Singh, Joe D. Joiner, Alba Herrero del Valle, Marleen Zwaagstra, Ida Jobe, Brian J. Ferguson, Frank J.M. van Kuppeveld, Yorgo Modis

**Affiliations:** 1 Molecular Immunity Unit, Department of Medicine, University of Cambridge, MRC Laboratory of Molecular Biology, Cambridge CB2 0QH, UK; 2 Cambridge Institute of Therapeutic Immunology & Infectious Disease (CITIID), Department of Medicine, University of Cambridge, Cambridge CB2 0AW, UK; 3 Department of Pathology, University of Cambridge, Cambridge CB2 1QP, UK; 4 Department of Genomes and Genetics, Institut Pasteur, 75724 Paris, France; 5 Section of Virology, Division of Infectious Diseases & Immunology, Department of Biomolecular Health Sciences, Faculty of Veterinary Medicine, Utrecht University, 3584 CL Utrecht, the Netherlands

## Abstract

MDA5 recognizes double-stranded RNA (dsRNA) from viruses and retroelements. Cooperative filament formation and ATP-dependent proofreading confer MDA5 with the necessary sensitivity and specificity for dsRNA. Many MDA5 genetic variants are associated with protection from autoimmune disease while increasing the risk of infection and chronic inflammation. How these variants affect RNA sensing remains unclear. Here, we determine the consequences of autoimmune-protective variants on the molecular structure and activities of MDA5. Rare variants E627* and I923V reduce the interferon response to picornavirus infection. E627* does not bind RNA. I923V is ATPase hyperactive, causing premature dissociation from dsRNA. Cryoelectron microscopy (cryo-EM) structures of MDA5 I923V bound to dsRNA at different stages of ATP hydrolysis reveal smaller RNA binding interfaces, leading to excessive proofreading activity. Variants R843H and T946A, which are genetically linked and cause mild phenotypes, did not affect cytokine induction, suggesting an indirect disease mechanism. In conclusion, autoimmune-protective MDA5 variants dampen MDA5-dependent signaling via multiple mechanisms.

## Graphical abstract

**Graphical abstract:**
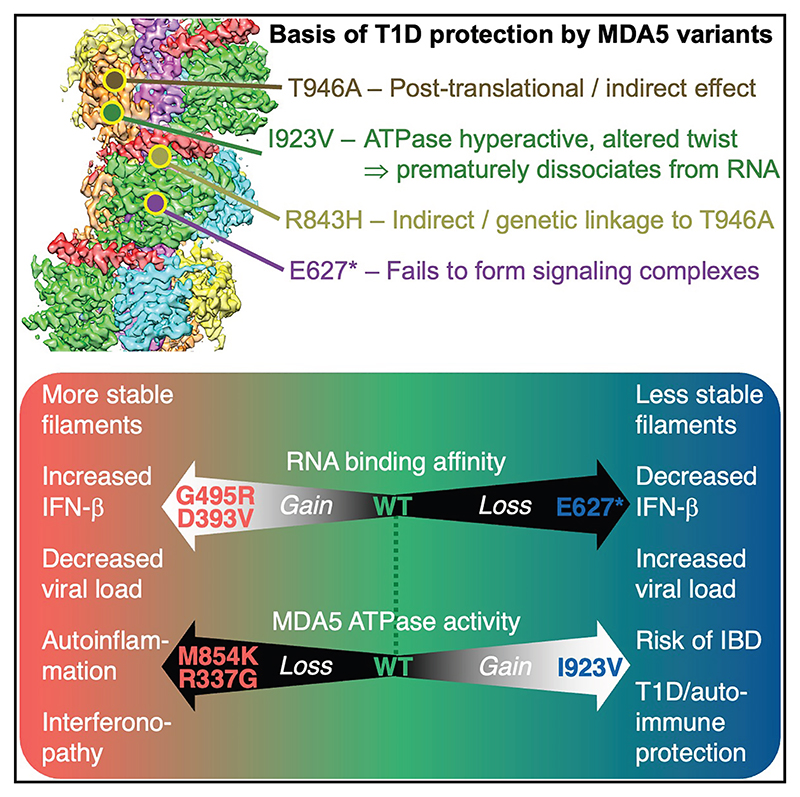

## Introduction

**Figure 1:**
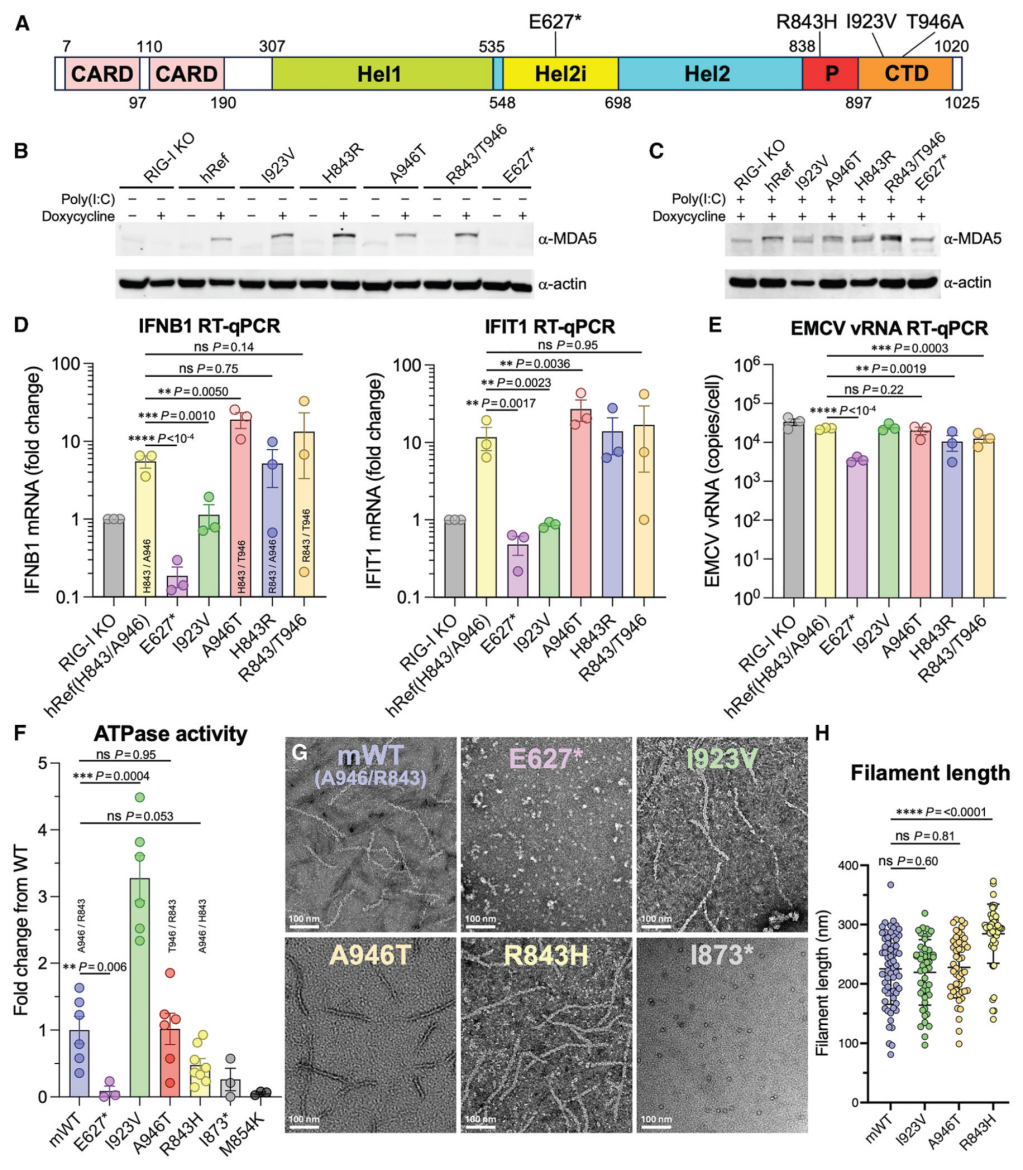
Effects of T1D-protective MDA5 variants on the antiviral interferon response and the ATPase and filament-forming activities of MDA5 (A) MDA5 domain organization. CARD, caspase recruitment domain; Hel1 and Hel2, RecA-like helicase domains; Hel2i, Hel2 insert domain; P, pincer domain; CTD, C-terminal domain. (B) Western blot showing doxycycline-inducible expression of human MDA5 variants in stable A549 RIG-I KO cell lines and the absence of endogenous MDA5 expression in the parent RIG-I KO cell line. (C) Western blot showing doxycycline-induced expression of MDA5 variants in the A549 cell lines after poly(I:C) stimulation. (D) RT-qPCR quantification of *IFNB1* and *IFIT1* mRNA in A549 RIG-I KO cells stably expressing the indicated human MDA5 variant under a doxycycline-inducible promoter 7 h after infection with encephalomyocarditis virus (EMCV). I923V and E627* inhibit the antiviral response. hRef, human reference sequence. Note that hRef MDA5 has H843; mouse MDA5 has R843. (E) RT-qPCR quantification of EMCV RNA 7 h post-infection. See also Figure S1. Error bars in (D) and (E) represent mean ± SEM (2 or 3 measurements from 3 independent experiments). (F) ATPase activities of mouse MDA5 variants, normalized to WT and with 0-fold change set to the ATPase activity of the M854K variant (t = 15 min). Error bars represent mean ± SEM (3 or 6 measurements from 1 or 2 independent experiments, respectively). Source data for (B)–(D) are provided as Data S1. mWT, mouse wild type. (G) Negative-stain electron micrographs of mouse MDA5 variants with 1-kb dsRNA. Each micrograph is representative of at least eight images. (H) Filament length measurements taken from electron micrographs shown in (G) and Data S1.

Viruses deliver or generate RNA in the cytosol. Cytosolic double-stranded RNA (dsRNA), one of the most proinflammatory molecular signals from viruses and retroelements,^1^ is sensed in vertebrates by RIG-I,^2,3^ MDA5,^3–5^ LGP2,^6–10^ and protein kinase R.^11,12^ These are complemented in mammals by the oligoadenylate synthases,^13,14^ ZBP1, and, in humans, NLRP1.^15^ MDA5 recognizes dsRNAs longer than 100 base pairs (bp)^4,5^ and is the primary innate immune sensor for many viruses, including SARS-CoV-2.^16,17^ MDA5 is a superfamily 2 helicase with two RecA-like domains (Hel1 and Hel2), an insert domain (Hel2i), and a C-terminal domain (CTD) linked to Hel2 by a pair of α helices known as the pincer domain (Figure 1A). These domains cooperatively bind dsRNA to form helical MDA5-dsRNA filaments.^18–23^ Filament formation induces the tandem N-terminal caspase recruitment domains (CARDs) of MDA5 to oligomerize.^18,19^ MDA5 CARD oligomers recruit MAVS (mitochondrial antiviral signaling protein) via CARD-CARD interactions, nucleating the assembly of MAVS CARD microfibrils,^24,25^ which function as supramolecular organizing centers for downstream effectors of the interferon-β (IFN-β) and nuclear factor κB (NF-κB) inflammatory responses.^4,5,24,26^

Viral dsRNA can be difficult to distinguish from endogenous RNA. Innate immune responses must be sensitive enough to detect infection and specific enough to avoid activation by cellular RNA. The ATPase activity of MDA5 confers the necessary specificity of dsRNA recognition. Conformational changes coupled to ATP hydrolysis fulfill a proofreading function by promoting the dissociation of MDA5 from endogenous dsRNAs,^22,23^ which are shorter and have weaker base pairing due to mismatches and A-to-I deamination by ADAR1.^27–29^ The cooperativity of both filament formation and ATP hydrolysis by MDA5 confers sensitivity by encoding greater stability for long MDA5 filaments such that only filaments formed on longer dsRNAs of viral origin persist long enough to activate signaling.^22,30^

The gene encoding MDA5, *IFIH1*, is a hotspot for natural variants with diverse clinical associations. Approximately 40 missense variants are associated with autoinflammatory disease, including Aicardi-Goutières syndrome (AGS) and Singleton-Merten syndrome (SMS).^31–34^ In most of these variants, the amino acid substitutions inhibit ATP hydrolysis, either directly (e.g., R337G)^33^ or allosterically (e.g., M854K).^23^ This disrupts ATP-dependent proofreading and allows MDA5 signaling complexes to form on endogenous dsRNAs.^23,33–36^ Other autoinflammatory variants map to the RNA binding interface and promote signaling from endogenous RNAs by increasing the RNA binding affinity of MDA5.^33^ A distinct set of variants reduces the risk of developing certain autoimmune diseases, most notably type 1 diabetes (T1D). Missense single-nucleotide polymorphisms (SNPs) resulting in the MDA5 variants E627* (rs35744605), R843H (rs3747517), I923V (rs35667974), and T946A (rs1990760) are associated with protection from T1D.^37–43^ The E627* and I923V variants are rare, while R843H and T946A are common. The T946A variant results from an A:T-to-G:C bp substitution. The T1D-protective G:C (Ala946) allele frequencies are 30%–50% in White people and 70%–80% in African and Asian people.^38–42^ Similarly, R843H results from a G:C-to-A:T bp substitution, and the A:T (H843) allele frequency is 30–40% in White people and Africans, and 70% in Asian people.^38–42^ Variants T946A and R843H are in strong linkage disequilibrium with each other,^38,39,44^ such that Ala946 is predominantly found with His843 and Thr946 with Arg843.^42,44,45^ We note that most human *IFIH1* reference sequences contain the alleles encoding Ala946/His843. Wild-type (WT) mouse MDA5 contains Ala946/Arg843.

Whereas the autoinflammatory MDA5 variants increase basal IFN-β signaling, T1D protection correlates with reduced MDA5-dependent signaling.^42,46,47^ MDA5 knockout (KO) non-obese diabetic (NOD) mice are fully protected from T1D-like disease, and heterozygous (*MDA5*^+/−^) mice expressing half of the WT level of MDA5 are significantly protected from disease.^48^ The E627* and I923V variants decreased IFN-β signaling in a cell-based luciferase reporter assay.^46^ Basal IFN-β transcription was slightly reduced in the T946A variant in human peripheral blood mononuclear cells (PBMCs),^42^ transfected human or mouse cells,^42,46,47^ and mice.^42^ The R843H variant had no effect on IFN-β transcription.^46^ This suggests that variants E627*, I923V, and T946A either reduce the intracellular MDA5 protein concentration or alter the biochemical properties of the protein. There have been conflicting reports regarding whether these variants are transcribed at different levels, but overall, there is no compelling evidence of statistically significant differences in transcript or protein concentration for any MDA5 missense variant.^39,42,46,49–51^ Hence, how the T1D-protective MDA5 variants alter dsRNA sensing by MDA5 remains unknown. However, there is a strong clinical link between T1D onset and recent infection with RNA viruses, in particular coxsackieviruses and other enteroviruses. Patients with T1D have more frequent enterovirus infections, which precede the appearance of prediabetic markers, including autoantibodies.^52^ MDA5 recognizes RNA from *Picornaviridae*, including enteroviruses,^4,53^ which have evolved mechanisms to suppress IFN-β transcription.^54,55^ MDA5-induced inflammation and cell death in the pancreas following rotavirus infection contribute to autoimmune destruction of pancreatic β cells.^56^ Conversely, MDA5 KO mice are protected from disease upon infection with a β cell-tropic coxsackievirus.^57^ Therefore, a plausible hypothesis is that MDA5-dependent IFN production and inflammation following viral infection can trigger autoimmune β cell killing.

Here, we examine the consequences of T1D-protective MDA5 substitutions on the structure and activities of MDA5. We show that variants E627*, I923V, and T946A reduce the IFN-β response to picornavirus infection. The E627* variant cannot bind RNA or form filaments. The I923V variant has increased ATPase activity and reduced filament stability. Cryoelectron microscopy (cryo-EM) structures of the I923V variant bound to dsRNA at different stages of ATP hydrolysis reveal that the Ile923 side chain regulates the conformational changes necessary for ATP hydrolysis. The T946A substitution does not affect RNA recognition, suggesting an indirect T1D protection mechanism. Hence, we have uncovered multiple loss-of-function pathways that lead to T1D protection via distinct molecular mechanisms.

## Results

### T1D-protective MDA5 variants impair type I IFN response to picornavirus infection

Most but not all previous studies report that T1D-protective MDA5 variants reduce IFN responses. Transient overexpression of the E627* and I923V variants in human or mouse cells reduced *IFNB1* and *IFIT1* transcription with or without dsRNA (poly(I:C)) stimulation^42,46,47^ or upon picornavirus infection,^42,56^ resulting in increased viral loads.^56^ Similarly, the T946A substitution reduced transcription of type I and type III IFN signature genes in transiently transfected HEK293T cells^42,47^ and knockin mice.^42^ The T946A substitution also promoted picornavirus replication and increased infection mortality in mice.^42^ However, the T946A substitution was reported in other studies to increase *IFNB1* and *IFIT1* transcription and reduce picornavirus replication in transiently transfected HEK293 cells^56^ and to increase IFN-λ transcription in human pancreatic islets infected with coxsackievirus.^58^ In a further study, the T946A substitution had no effect on *IFNB1* transcription in transiently transfected mouse embryonic fibroblasts.^46^ Regarding the R843H variant, most studies conclude that its association with T1D protection is explained by its co-occurrence in most human subjects with the T946A variant,^38,42,46^ but one study reported that the His843/Thr946 variant (which rarely occurs in humans) increased type I and type III IFN transcription in HEK293 cells.^47^ To clarify the effects of these T1D-protective variants on MDA5-dependent IFN signaling, we generated RIG-I KO A549 human lung epithelial cell lines stably expressing each MDA5 variant under a doxycycline-inducible promoter (Figure 1B). RIG-I KO A549 cells have no basal endogenous MDA5 expression, only low levels of endogenous MDA5 expression following stimulation with poly(I:C) dsRNA (Figure 1C), and there was no poly(I:C)-induced *IFNB1* transcription in these cells.^59^ Cells were infected with encephalomyocarditis virus (EMCV), a model picornavirus. We used a recombinant EMCV containing mutations in its Leader protein, i.e., the viral IFN antagonist protein,^55^ to achieve strong type I IFN transcription signals. *IFNB1* and *IFIT1* transcription, as well as EMCV RNA replication, were quantified by RT-qPCR 7 h after infection. We found that the E627* and I923V variants both failed to induce a type I IFN response (Figures 1D, S1A, and S1C). Cells expressing I923V MDA5 had the same *IFNB1* and *IFIT1* transcription levels as the untransduced reference cell line. The E627* variant showed a further reduction in *IFNB1* transcription and, to a lesser extent, *IFIT1* transcription, below the levels in the reference cell line. This was not due to increased cell death, as transcription levels were normalized to *Actin* expression. We note that the E627* variant was expressed at a much lower level than the other variants (Figure 1B). The human reference variant (H843/A946) induced *IFNB1* and *IFIT1* transcription to the same extent as the common R843/T946 variant and the rare R843/A946 (H843R) variant, but the rare H843/T946 (A946T) variant increased *IFNB1* and *IFIT1* transcription (Figure 1D), consistent with a previous study.^47^ None of the variants significantly altered baseline *IFNB1* or *IFIT1* transcription in the absence of EMCV infection (Figures S1B and S1D). EMCV replicated efficiently in all cell lines (Figures 1E and S1E). Hence, the effects of the variants on *IFNB1* and *IFIT1* transcription were not due to differences in viral RNA replication or viral dsRNA availability. In summary, our data show that the rare T1D-protective E627* and I923V variants cause loss of MDA5-dependent IFN-β signaling, in agreement with previous studies, whereas the protective R843H and T946A variants had no significant effect on EMCV-induced signaling in the combination commonly found in human subjects (H843/A946).

### ATPase and filament-forming activities of T1D-protective MDA5 variants

To assess the effects of T1D-protective substitutions on the biochemical properties of MDA5, we purified recombinant mouse WT, E627*, I923V, R843H, and A946T MDA5 proteins. The E627* protein lacked ATPase activity (Figure 1F) and did not form filaments on 1-kb dsRNA, based on negative-stain electron microscopy imaging (Figure 1G). The R843H variant appeared to have a slightly reduced ATPase activity and form slightly longer filaments than WT MDA5, although the reduction of ATPase activity was not statistically significant (Figures 1F–1H). In stark contrast, the I923V variant had 3.3-fold higher ATPase activity than WT MDA5 (Figure 1F), although filament formation was unaffected (Figures 1G and 1H). The A946T variant was notable in that its ATPase and filament-forming activities were the same as WT MDA5 (Figures 1F and 1G). We note that the A946T variant was previously reported to have reduced ATPase activity,^60^ but we observed the same ATPase activity for A946T and WT purified recombinant mouse MDA5. We conclude that T1D-protective substitutions have pleiotropic effects on the biochemical activities of MDA5 and therefore act via distinct mechanisms. E627* is a simple loss-of-function mutant that lacks signaling activity because it cannot bind dsRNA. We note that truncation of the CTD (I873*) was sufficient to abrogate both ATPase activity and filament formation on dsRNA (Figures 1F and 1G). The loss of signaling activity of the I923V variant may be explained by its increased ATPase activity because ATP hydrolysis promotes dissociation from dsRNA.^22,23^ This is the converse of substitutions that inhibit ATP hydrolysis without affecting filament formation, which reduce dissociation of MDA5 signaling complexes from dsRNA, including endogenous dsRNAs, and hence trigger autoinflammatory signaling.^23,33^

### T1D-protective MDA5 variants have reduced affinity for dsRNA

**Figure 2:**
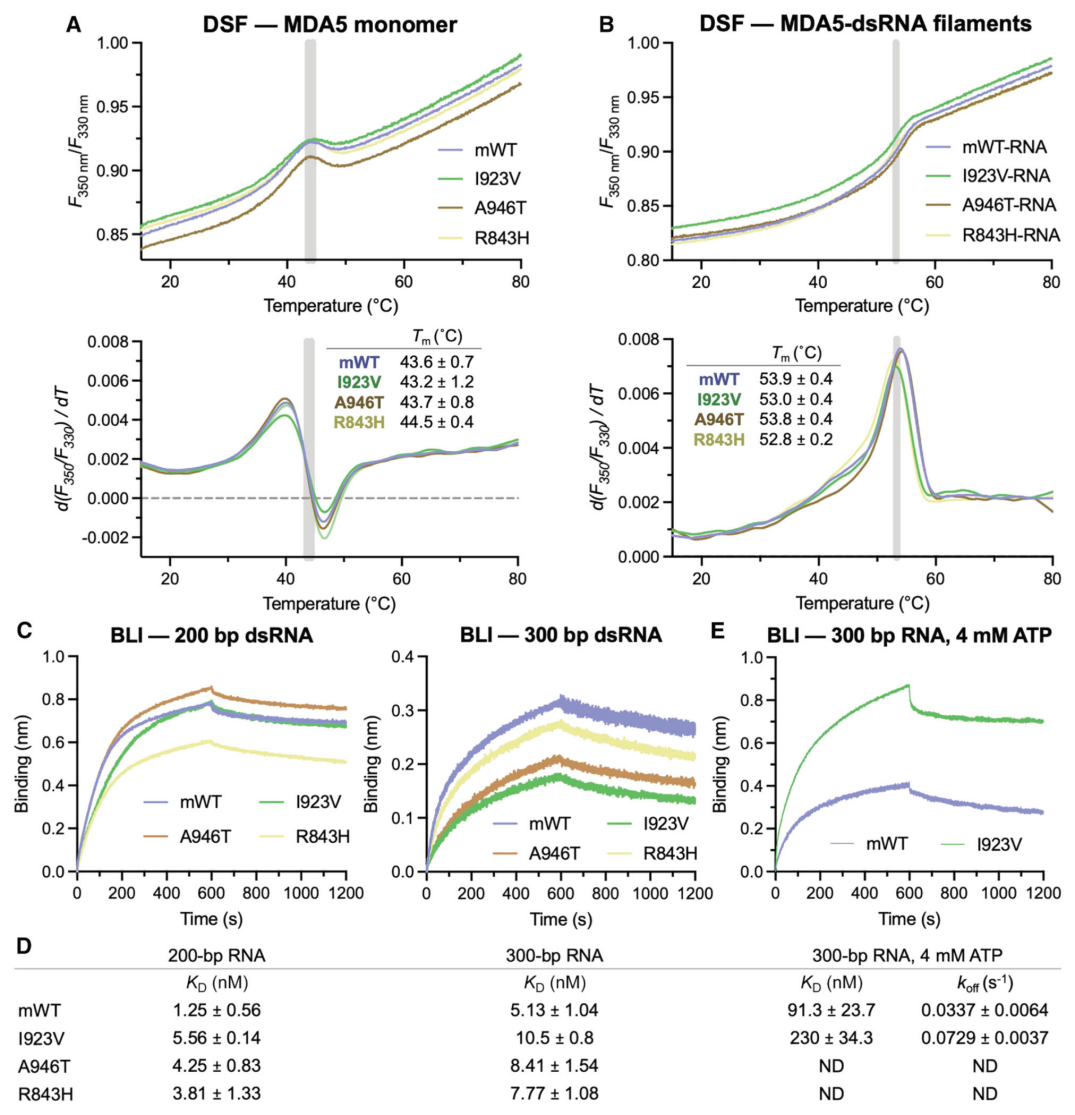
Thermostability and RNA binding affinities of T1D-protective MDA5 variants (A) Differential scanning fluorimetry (DSF) of WT and variant MDA5 proteins. Intrinsic protein fluorescence at 330 and 350 nm was measured and the fluorescence ratio plotted as a function of temperature. Gray lines indicate the melting temperatures (*T*_m_) of the variants. (B) The *T*_m_ of the MDA5 variants was higher in the presence of 1-kb dsRNA. *T*_*m*_ values in (A) and (B) are the mean ± SD from at least 8 replicates, and curves show the average of all replicates. (C) Bio-layer interferometry (BLI) with 3′-biotinylated dsRNA immobilized on a streptavidin biosensor and 125 nM mouse MDA5 in the mobile phase (see also Figure S2). Curves from a single representative experiment are shown. (D) Dissociation constants (K_D_ values) derived from the curves in (C). The uncertainties are the SEM from at least three independent experiments. (E) BLI curves (from a single representative run) for binding of mouse MDA5 to 300-bp dsRNA, with the addition of ATP in the dissociation step. K_D_ values and dissociation rate constants (*k*_off_) are shown in (D) with the SEM calculated from at least four independent experiments.

The ATPase hyperactivity of the I923V variant suggests that the I923V substitution promotes dissociation from dsRNA. To quantify this effect, we used differential scanning fluorimetry (DSF) and biolayer interferometry (BLI) with purified recombinant mouse MDA5 proteins. The WT, I923V, A946T, and R843H variants had the same thermostability based on their DSF melting curves. These variants were significantly more thermostable under filament-forming conditions than as monomers in solution, with the melting temperature increasing by 9°C–10°C following the addition of 1-kb dsRNA (Figures 2A and 2B). In a more direct measure of binding, we used BLI. Although the cooperative binding of MDA5 to dsRNA results in a complex binding model, we could determine overall apparent dissociation constants (K_D_ values) for MDA5 binding to 200-bp and 300-bp dsRNA. Based on the K_D_ values, the I923V, A946T, and R843H variants had slightly (2- to 4-fold) lower affinities for 200- and 300-bp dsRNA than WT MDA5 (Figures 2C and 2D), while the E627* and I873* mutants failed to bind dsRNA (Figures S2A and S2B). The same trend was observed with 1-kb dsRNA, but K_D_ values could not be accurately determined due to the complex multiphasic shapes of the BLI curves observed with the longer dsRNA (Figures S2C–S2E).

Once bound to dsRNA, the MDA5 variants tested dissociated very slowly, such that dissociation rate constants (*k*_off_) could not be accurately determined. To assess whether the increased ATPase activity of the I923V variant correlated with increased dissociation from dsRNA, we performed BLI experiments with the addition of ATP at the beginning of the dissociation phase. This caused both WT and I923V variants to dissociate from dsRNA significantly faster, allowing off rates to be calculated (Figures 2D and 2E). The I923V variant displayed a 2-fold greater dissociation rate than WT MDA5 in the presence of ATP (0.0729 versus 0.0337 s^−1^).

We conclude that the E627* truncation causes complete loss of function, consistent with its lack of filament-forming activity, whereas the I923V substitution significantly increases ATPase activity, which promotes dissociation from dsRNA and hence reduces the stability of signaling complexes. The A946T and R843H substitutions appeared to slightly reduce dsRNA binding affinity, but the significance of this observation remains unclear, given that these substitutions did not affect ATPase activity and filament formation in a manner consistent with a reduction in dsRNA binding affinity.

### I923V MDA5 has an altered distribution of conformational states during catalysis

**Figure 3:**
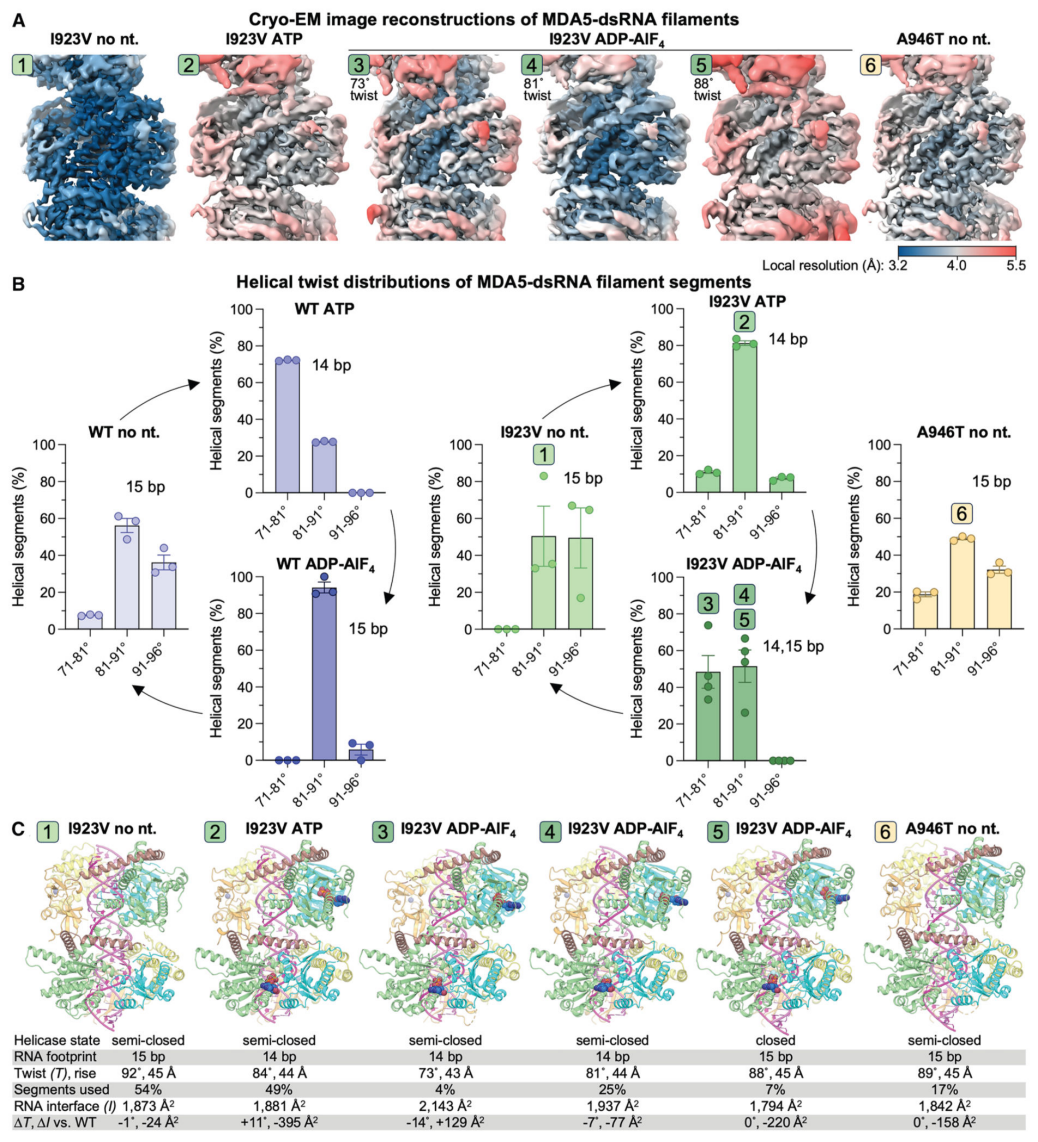
Cryo-EM structures of I923V and A946T MDA5 bound to dsRNA at different stages of ATP hydrolysis (A) Cryo-EM image reconstructions of MDA5-dsRNA filaments with helical symmetry averaging. (B) Helical twist distributions of MDA5-dsRNA filament segments after 3D classification. Error bars represent mean ± SEM from 3 classifications. (C) Atomic models and their structural parameters. Two helical subunits are shown for each model. See Figure S3 for Fourier shell correlation curves.

Structural studies have shown that MDA5 variants associated with autoinflammatory disease impair discrimination of endogenous RNA from viral RNA by altering the conformational changes that are coupled to the ATPase cycle, directly or indirectly.^23^ To gain a mechanistic understanding of the effects of the I923V and A946T substitutions on MDA5 function, we determined cryo-EM structures of mouse MDA5-dsRNA filaments containing each of these substitutions at different stages of ATP hydrolysis (Figure 3A). Four cryo-EM datasets were collected: I923V MDA5 filaments with ATP, transition-state analog ADP-AlF_4_, or no nucleotide bound and A946T filaments without nucleotide (Table S1; Figure S3). Three-dimensional (3D) classification of the maps with helical symmetry averaging applied allowed us to analyze the helical twist distributions of the filament segments for each dataset. We found that upon binding ATP, filaments formed from the I923V variant mostly had an intermediate helical twist (81°–91°) instead of the low twist (71°–81°) observed in ATP-bound WT MDA5 filaments (Figure 3B). Moreover, with ADP-AlF_4_ bound, the I923V MDA5 filaments had a broad distribution of low to intermediate twists instead of the narrow distribution of intermediate twists observed in the transition state of WT MDA5. In the absence of nucleotide, the I923V and A946T variants both had similar twist distributions to WT MDA5, with intermediate to high twists (81°–96°; Figure 3B). Hence, the I923V substitution alters the helical twist distributions of MDA5-dsRNA filaments in the ATP-bound and ADP-AlF_4_-bound states.

Processing and refinement of our four cryo-EM datasets yielded six maps with resolutions sufficient to build and refine atomic models of the protein, dsRNA, and bound nucleotides. The ATP-bound I923V dataset and nucleotide-free I923V and A946T datasets each yielded one map, whereas the ADP-AlF_4_-bound I923V dataset yielded three different maps (Figure 3A). The atomic models of the nucleotide-free states of the I923V and A946T variants are similar to previously reported nucleotide-free structures of WT MDA5,^22^ with 15-bp RNA footprints, intermediate to high helical twists (89°–92°), and protein-RNA interaction areas of 1,800–1,900 Å^2^ per MDA5 subunit calculated with PISA^61^ (Figure 3C). The ATP-bound I923V structure has the same 14-bp RNA footprint as ATP-bound WT MDA5^22^ but a significantly higher helical twist (84°, versus 73° for WT) and a 20% smaller protein-RNA interaction area (Figure 3C). The three ADP-AlF_4_-bound I923V structures have helical twists of 73°, 81°, and 88°, respectively, reflecting the helical twist distribution of ADP-AlF_4_-bound I923V filaments (Figure 3B). The 73°-twist and 81°-twist structures have 14-bp RNA footprints, while the 88°-twist structure has a 15-bp footprint. The 88°-twist structure is similar to the ADP-AlF_4_-bound WT MDA5 structure, albeit with a 10% smaller protein-RNA interaction area (Figure 3C). However, the 73°-twist and 81°-twist structures both differ from the WT transition-state structure in that they retain the same 14-bp footprint and lower twist as the ATP-bound ground state. Additionally, only in the 88°-twist structure are the Hel1 and Hel2 domains in the catalytically competent closed conformation (as defined based on the Hel1-Hel2 rotation angle; see STAR Methods). Indeed, the 73°-twist and 81°-twist structures are in the semi-closed conformation, in which the Hel2 domain is not engaged with the nucleotide. Together, the structural features of the 73°-twist and 81°-twist ADP-AlF_4_-bound I923V structures indicate that they do not represent the catalytic transition state but rather intermediates that more closely resemble the ground state. In summary, the I923V and A946T variants adopt similar sets of structures as WT MDA5, but the I923V variant has a smaller protein-RNA interaction area than WT in the ATP-bound state, and I923V can accommodate ADP-AlF_4_ in its active site in a ground-state-like conformation with a 14-bp RNA footprint as well as in the closed transition state with a 15-bp footprint.

### Isoleucine is required at position 923 to sterically regulate ATPase activity

**Figure 4:**
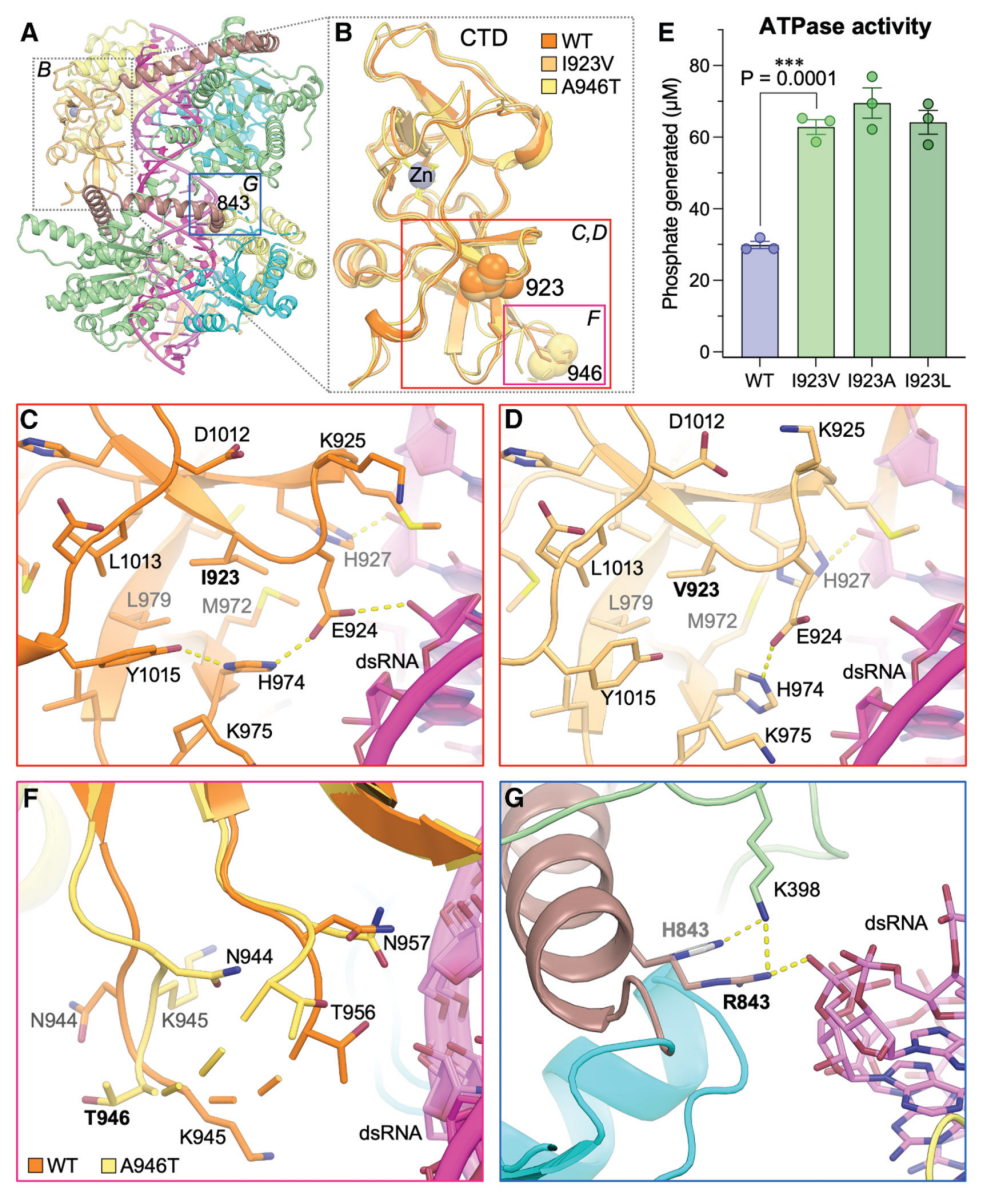
Isoleucine is required at position 923 to sterically regulate ATPase activity (A) Overall structure of the MDA5-dsRNA filament without nucleotide. (B) The superimposed C-terminal domains of WT, I923V, and A946T MDA5 from the cryo-EM structures without nucleotide (PDB: 6H61, 9F0J, and 9F3P). (C) Closeup of Ile923 and surrounding residues in the WT MDA5 structure. (D) Closeup of Val923 and surrounding residues in the MDA5 I923V structure. (E) ATPase activities of WT, I923V, I923A, and I923L MDA5. Error bars represent mean ± SEM (3 measurements from one experiment). (F) Closeup of the loop containing residue 946 in the superimposed structures of WT and A946T MDA5. (G) Closeup of the loop containing residue 843 in the structure of A946T MDA5. The arginine side chain is shown (red), along with a modeled histidine side chain (gray) to represent the R843H substitution.

Closer examination of the structures of the I923V and A946T variants did not reveal any noteworthy changes in the fold of the CTD, where both substitutions are located (Figures 4A and 4B). In all available structures, the side chain of residue 923 contributes to the hydrophobic core of the CTD and forms multiple van der Waals contacts with surrounding side chains. In the structures of the I923V variant, there are minor differences in the side-chain positions of several nearby residues that occur, including E924, K925, H974, and Y1015, possibly to compensate for the smaller size of the valine side chain in the hydrophobic core (Figures 4C and 4D). As a result, a hydrogen bond between the side chain of E924 and a ribose hydroxyl group in the RNA present in the nucleotide-free structure of WT MDA5 is lost in the I923V nucleotide-free structure. The loss of this protein-RNA contact partly explains the reduced RNA binding affinity and protein-RNA interaction area of the I923V variant reported above.

Considering the 3-fold increase in ATPase activity associated with relatively subtle structural changes in the I923V variant, we measured the ATPase activities of MDA5 proteins with slightly more or less conservative mutations at position 923, substituting either alanine or leucine for isoleucine. We found that the I923A and I923L mutants had the same ATPase activity as I923V (Figure 4E). Hence, further decreasing the bulk of the hydrophobic side chain from valine to alanine did not further increase ATPase activity, and substitution with leucine, an isomer of the isoleucine residue found in WT MDA5, did not reduce ATPase to the level of WT MDA5. We conclude that the isoleucine side chain at position 923 regulates the conformational changes necessary for ATP hydrolysis by shaping the CTD fold to tune ATPase activity to an optimal evolved level in WT MDA5.

In contrast to residue 923, residue 946 is located within a partly disordered solvent-exposed loop that does not contribute to the core fold of the CTD. This loop is poorly defined in the cryo-EM maps. There are minor differences in the conformation of the loop in the WT and A946T structures, but these differences do not alter the protein fold, RNA binding interface, or active site (Figure 4F). This is consistent with the absence of any significant effects of the A946T substitution on the overall structure, filament assembly, and ATPase activity of MDA5. Together, our data suggest that the A946T substitution has no direct effect on the structural or biochemical properties of MDA5. We note that threonine is a phosphorylatable residue, raising the possibility that T1D protection associated with the T946A allele may be due to a difference in phosphorylation state. Alternatively, the T946A allele may be non-causal and its association with T1D protection attributable to its linkage with the R843H allele. Notably, in a subset of the cryo-EM structures, the R843 side chain forms a salt bridge with the dsRNA phosphate backbone, and the R843H substitution would result in loss of this salt bridge (Figure 4G).

## Discussion

We have shown here that T1D-protective MDA5 variants have pleiotropic effects on the structural and biochemical activities of MDA5 (Figure S4). The rare variants E627* and I923V reduce the MDA5-dependent cellular IFN-β response to picornavirus infection, in agreement with previous studies, but we found that variants R843H and T946A, in combination, as they are predominantly found in humans due to linkage disequilibrium, had no significant effect on IFN-β expression. For the E627* variant, the deletion of most of the helicase module of MDA5 is sufficient to explain the loss of RNA binding, destabilization of the protein fold, and, therefore, the loss of signaling function. In contrast, we find that the I923V variant has increased ATPase activity. Our cryo-EM structures of the I923V variant bound to dsRNA at different stages of ATP hydrolysis reveal that the Ile923 side chain regulates the conformational changes necessary for ATP hydrolysis. The increased ATPase activity of the I923V variant may be attributed to the slight reduction of the surface complementarity of the MDA5 RNA binding interface in the I923V variant. As a result, the I923V variant has altered helical twist distributions of MDA5-dsRNA filaments in the ATP-bound and ADP-AlF_4_-bound states. Since engineered I923A and I923L variants had the same hyperactive ATPase activity as I923V, we propose that the ancestral isoleucine side chain at position 923 functions as a molecular brake, regulating the conformational changes necessary for ATP hydrolysis by shaping the CTD fold to tune ATPase activity to an optimal evolved level in WT MDA5. The increased ATPase activity of the I923V variant will result in overzealous proofreading, promoting premature dissociation from dsRNA. This, in turn, restricts the formation of active signaling complexes on dsRNA.

The A946T substitution had no apparent effect on ATPase activity, structure, or filament formation on dsRNA in our assays, leaving the molecular basis of T1D-protective effects of this mutant unclear. The linked R843H substitution appeared to slightly reduce ATPase activity and increase filament length. However, by analogy with ATPase-deficient variants, these effects would be expected to be associated with increases in dsRNA binding affinity, signaling activity, and autoinflammation, none of which were observed for the R843H variant. On the contrary, we found that the R843H substitution slightly reduced dsRNA binding affinity. A new meta-analysis of recent, large genome-wide association study (GWAS) datasets confirmed the association of E627*, I923V, T946A, and R843H with significant levels of protection against T1D as well as other autoimmune-related diseases, including psoriasis and hypothyroidism.^62^ Fine-mapping suggested that the effect associated with the R843H variant was not independent and was likely a result of linkage with the T946A variant,^62^ consistent with previous studies concluding that associations with R843H could be explained by its co-occurrence with T946A.^38,42^ Together, the available evidence and analyses suggest that R843H is not independently protective against T1D and that T946A is protective via an indirect mechanism. We hypothesize that a threonine residue at position 946 can be phosphorylated or otherwise post-translationally modified to promote signaling. For example, a phosphothreonine at position 946 may contribute to the recruitment of signaling cofactors (e.g., PACT, ZCCHC3, TRIM65, or K63-linked ubiquitin chains) or, alternatively, increase the lifetime of MDA5 in the cytosol (e.g., by protecting it from degradation). However, there is no experimental evidence to support the phosphorylation at T946.

In conclusion, the T1D-protective MDA5 variants E627*, I923V, and T946A each ultimately lead to a loss of MDA5-dependent signaling but do so via three distinct mechanisms. The net loss-of-function effect that T1D-protective mutations have on IFN signaling is the converse of MDA5 variants associated with autoinflammatory disease, which have a gain-of-function effect on signaling and also act via multiple distinct mechanisms.^23,33^ Notably, in addition to protecting against autoimmune-related diseases, these loss-of-function variants increased the risk of viral infection and inflammatory bowel disease (IBD), specifically Crohn’s disease and ulcerative colitis.^62^ Furthermore, the degree of T1D protection and IBD risk were closely correlated, suggesting that MDA5 loss-of-function variants offer a fundamental fitness trade-off between viral clearance and tissue damage.^62^ Robust clinical links have emerged between enteric virus infection and the onset of both T1D^52,63^ and IBD.^64^ Indeed, MDA5-induced inflammation and cell death in the pancreas following rotavirus infection contribute to autoimmune destruction of pancreatic β cells,^56^ and gastrointestinal infection has been associated with increased risk of developing IBD in a large clinical study.^64^ We propose a model in which loss-of-function MDA5 variants protect against T1D by reducing autoimmune β cell killing triggered by MDA5-dependent IFN-β production and inflammation following viral infection while also contributing to the induction of IBD^62,64^ due to increased susceptibility to viral infection from the loss of MDA5 antiviral activity.

### Limitations of the study

The signal-to-noise ratio in the cell infection assays measuring MDA5-dependent IFN induction following EMCV infection may be limited by the induction of endogenous MDA5 expression in the parent A549 cells line (A549 *RIG-I*^−/−^ *ACE2*^−/−^), such that some of the *IFNB1* induction observed could be due to endogenous MDA5 rather than from the overexpressed transduced MDA5 variant. However, we showed previously that MDA5 expression is not significantly induced during infection by EMCV, which is particularly effective at suppressing the IFN response,^54,55^ and that stimulation with poly(I:C) dsRNA did not induce *IFNB1* expression in A549 RIG-I KO cells.^59^ Furthermore, western blot data showed that levels of endogenous MDA5 remained low compared to the overexpressed variants, following poly(I:C) stimulation (Figures 1B and 1C).

The interpretation of the BLI data for MDA5 binding to dsRNA, and of dsRNA-binding data in general, is limited by the highly cooperative nature of MDA5 binding to dsRNA. Cooperative filament formation by MDA5 means that dsRNA binding follows a complex, multistep pathway that, in some cases, cannot be approximated by a simple binding model. This prevented K_D_ and *k*_off_ values from being calculated for longer (1-kb) dsRNA ligands from BLI data, although the binding curves for shorter (200- to 300-bp) dsRNA could still be approximated by a simple binding model for kinetic and equilibrium constants to be calculated with sufficient accuracy. The large and dynamic nature of MDA5-dsRNA complexes also hampered attempts to determine *K*_M_ or K_D_ values for ATP from ATPase activity assay or isothermal titration calorimetry data, respectively.

## Resource Availability

### Lead contact

Requests for further information and resources should be directed to and will be fulfilled by the lead contact, Yorgo Modis (ymodis@mrc-lmb.cam.ac.uk).

### Materials availability

All unique and stable reagents generated in this study are available from the lead contact with a completed materials transfer agreement.

### Star★Methods

Detailed methods are provided in the online version of this paper and include the following:

- KEY RESOURCES TABLE
- EXPERIMENTAL MODEL DETAILS
  ○ Cell lines and microbe strains
- METHOD DETAILS
  ○ RNA synthesis
  ○ Generation of cell lines expressing MDA5 T1D-protective variants
  ○ Cell culture and induction
  ○ Immunoblotting to measure MDA5 expression
  ○ Virus infection assays
  ○ MDA5 protein purification
  ○ Nanoscale differential scanning fluorimetry (nanoDSF)
  ○ Bio-layer interferometry (BLI)
  ○ ATPase assay
  ○ Negative stain EM
  ○ Cryo-EM sample preparation and data collection
  ○ Image processing and helical reconstruction
  ○ Model building and refinement
- QUANTIFICATION AND STATISTICAL ANALYSIS

## Star★Methods

### Key Resources Table

**Table:** 

REAGENT or RESOURCE	SOURCE	IDENTIFIER
Antibodies		
Rabbit polyclonal anti-MDA5	Enzo Life Sciences	Cat#ENZ-ABS299; LOT M44CZ27; RRID: AB_2893162;
Mouse monoclonal anti-FLAG	Sigma-Aldrich	Cat# F1804; RRID: AB_262044
Mouse anti-beta actin	Antibodies.com	Cat#A85272; LOT 51008; RRID AB_2748874
Goat anti-mouse IgG (H + L) (DyLight 680 Conjugate)	Cell Signaling Technology	Cat#5470; LOT 15; RRID:AB_10696895
Goat anti-Mouse IgG (H + L) DyLight™ 680	Invitrogen	Cat#35519; LOT WJ330986; RRID: AB1965956
Goat anti-rabbit IgG (H + L) (DyLight 800 Conjugate)	Cell Signaling Technology	Cat#5151; LOT 15; RRID:AB_10697505
Bacterial and virus strains		
*Escherichia coli* Rosetta(DE3)pLysS strain	Novagen	Cat#71403
EMCV Mengovirus strain	Hato et al.^55^	N/A
Chemicals, peptides, and recombinant proteins		
Recombinant proteins: MDA5 variants	This paper	N/A
Polybrene	Tocris	Cat#7711
Dulbecco’s modified Eagle’s medium (DMEM)	Gibco	Cat#31966047
Fetal Bovine Serum (FBS)	Gibco	Cat#10270106
cOmplete EDTA-free protease inhibitor cocktail	Roche	Cat#11873580001
PhosSTOP	Roche	Cat#04906845001
Salt Active Nuclease	Merck	Cat#SRE0015
TURBO™ DNase	Invitrogen	Cat#AM2238
Lipofectamine™ MessengerMAX™ Transfection Reagent	Invitrogen	Cat#LMRNA003
Opti-MEM Medium	Gibco	Cat#31985070
Poly(I:C) HMW long synthetic dsRNA analog	InvivoGen	Cat#tlrl-pic
Critical commercial assays		
Q5 Site-Directed Mutagenesis Kit	New England BioLabs	Cat#E0554S
HiScribe T7 High Yield RNA Synthesis Kit	New England BioLabs	Cat#E2040S
MEGAscript T7 Transcription Kit	Invitrogen	Cat#AM1333
PureLink RNA Mini Kit	ThermoFisher	Cat#12183018A
Monarch RNA Cleanup Kit	New England BioLabs	Cat#T2050L
Lenti-X Packaging Single Shots	Takara	Cat#631275
Lenti-X p24 Rapid Titer (Single Wash) Kit	Takara	Cat# 631476
Pierce RNA 3′ End Biotinylation Kit	ThermoFisher	Cat#20160
ATPase/GTPase Activity Assay Kit	Sigma-Aldrich	Cat#MAK113
Deposited data		
EM data: mMDA5 I923V (ATP)	This paper	EMDB: EMD-50165
EM data: mMDA5 I923V (ADP-AlF_4_) 73° twist	This paper	EMDB: EMD-50150
EM data: mMDA5 I923V (ADP-AlF_4_) 81° twist	This paper	EMDB: EMD-50136
EM data: mMDA5 I923V (ADP-AlF_4_) 88° twist	This paper	EMDB: EMD-50137
EM data: mMDA5 I923V (no nt.)	This paper	EMDB: EMD-50111
EM data: mMDA5 A946T (no nt.)	This paper	EMDB: EMD-50175
Atomic model: mMDA5 I923V (ATP)	This paper	PDB: 9F2W
Atomic model: mMDA5 I923V (ADP-AlF_4_) 73° twist	This paper	PDB: 9F2L
Atomic model: mMDA5 I923V (ADP-AlF_4_) 81° twist	This paper	PDB: 9F1U
Atomic model: mMDA5 I923V (ADP-AlF_4_) 88° twist	This paper	PDB: 9F20
Atomic model: mMDA5 I923V (no nucelotide)	This paper	PDB: 9F0J
Atomic model: mMDA5 A946T (no nucelotide)	This paper	PDB: 9F3P
Experimental models: Cell lines		
Human: HEK293T cells	ATCC	RRID: CVCL_0063
Hamster: BHK-21 fibroblasts	ATCC	RRID: CVCL_1915
Human: A549 RIG-I^−/ −^cells	Teague et al.^59^	N/A
Human: A549 RIG-I^−/ −^ ACE2^+^ cells	This paper	N/A
Recombinant DNA		
Plasmid: pLVX-TetOne-Puro	Takara	Cat#631849
Plasmid: pLVX-TetOne-Puro-hMDA5	This paper	N/A
Plasmid: pET28a(+)	SigmaAldrich	Cat#69864
Software and algorithms		
Octet Analysis Studio v11.1	Sartorius	sartorius.com
Prism v10	GraphPad	graphpad.com
PR.Stability Analysis v1.1	NanoTemper	nanotempertech.com
EPU	ThermoFisher	thermofisher.com
SerialEM	Mastronarde et al.^67^	bio3d.colorado.edu
MotionCor2.0	Kimanius et al.^68^	N/A
Relion4.0	He et al.^70^	relion.readthedocs.io
CtfFind4.1	Rohou et al.^69^	N/A
crYOLO	Wagner et al.^71^	cryolo.readthedocs.io
UCSF Chimera	Pettersen et al.^73^	www.cgl.ucsf.edu/chimera
COOT	Emsley et al.^74^	www2.mrc-lmb.cam.ac.uk/personal/pemsley/coot/
Phenix 1.21	Liebschner et al.^75^	www.phenix-online.org
PISA server	Krissinel et al.^61^	ebi.ac.uk/pdbe/prot_int
Fiji v2.16	Schindelin et al.^66^	https://fiji.sc
Other		
QUANTIFOIL R1.2/1.3 300-mesh gold grids	Agar Scientific	Cat#AGS143-8
HisTrap HP nickel-affinity column	Cytiva	Cat#17-5248-02
Octet SA Biosensors	Sartorius	Cat#18-5019
Standard capillaries	NanoTemper	Cat#PR-C002

### Experimental Model Details

#### Cell lines and microbe strains

A549 cells originate from the lung tissue of a 58-year-old white male patient with lung adenocarcinoma (atcc.org). A549 cells were grown at 37°C in 5% CO_2_ in Dulbecco’s modified Eagle’s medium (DMEM), high glucose, with GlutaMAX Supplement and sodium pyruvate (Gibco; cat. no. 31966047), supplemented with 10% fetal bovine serum (FBS; Gibco). A549 RIG-I^−/−^ cell line was generated as previously described.^59^

Human embryonic kidney 293T (HEK293T) cells originate from kidney tissue from a healthy female fetus (atcc.org, RRID: CVCL_0063). HEK293T cells were grown at 37°C in 5% CO_2_ in high glucose DMEM with GlutaMAX Supplement (Gibco).

Baby Hamster Kidney (BHK-21) fibroblasts originate from healthy kidney tissue from a male golden hamster, *Mesocricetus auratus* (atcc.org, RRID:CVCL_1915). BHK-21 cells were grown at 37°C in 5% CO_2_ in high glucose DMEM supplemented with 10% fetal bovine serum (FBS), penicillin (100 U ml^−1^) and streptomycin (0.1 mg mL^−1^).

Recombinant encephalomyocarditis virus (EMCV), strain Mengovirus carrying mutations C19A and C22A in the zinc finger domain of the viral Leader protein (Leader-Zn mutant) were obtained from pM16.1 cDNA clones by oligonucleotide-directed mutagenesis as described previously.^55^

*Escherichia coli* Rosetta(DE3)pLysS cells were obtained from Novagen (cat. no. 71403).

### Method Details

#### RNA synthesis

RNAs were transcribed *in vitro* using the MEGAscript T7 Transcription Kit (Invitrogen, cat. no. AM1333) or HiScribe T7 High Yield RNA Synthesis Kit (New England BioLabs, cat. no. E2040S) following the manufacturers’ protocols. The 200-bp, 300-bp, and 1-kb dsRNA contained the first 200, 300 or 1000 bases of the mouse *IFIH1* gene, respectively. The complementary RNA strands were transcribed from DNA templates with a preceding 5′ TAATACGACTCACTATAG 3′ sequence and a T7 promoter on the coding strand. The *in vitro* transcription reactions were performed at 37°C for 2, 4, 6 h or overnight. Transcripts were treated with Turbo DNase and purified with the PureLink RNA Mini Kit (ThermoFisher, cat. no. 12183018A) or the Monarch RNA Cleanup Kit (500 μg) (New England BioLabs, cat. no. T2050L). Samples were eluted in nuclease-free duplex annealing buffer (30 mM HEPES pH 7.5, 100 mM KCl (Integrated DNA Technologies)). Eluted transcripts were incubated at 95°C for 5 min and cooled to room temperature over 2 h to eliminate secondary structure and enable annealing of complementary strands of RNA.

#### Generation of cell lines expressing MDA5 T1D-protective variants

RIG-I KO A549 cells expressing ACE2 (A549 RIG-I^−/−^ ACE2^+^ cells) were transduced with a recombinant lentivirus to express T1D-protective MDA5 variants in a doxycycline-dependent manner. To generate the lentiviruses, HEK293T cells grown in high glucose DMEM with GlutaMAX Supplement (Gibco) were transfected with a pLVX-TetOne-Puro vector (Takara) containing a gene encoding human MDA5 (UniProt: Q9BYX4) with an N-terminal FLAG tag. HEK293T cells were transfected (24 h after seeding 4-5 x10^6^ cells in 8 mL of medium in a 10-cm plate) with 7.0 μg of pLVX-TetOne-Puro-hMDA5 vector in 600 μL sterile water with Lenti-X Packaging Single Shots (Takara). 16 h post-transfection, 6 mL of fresh complete growth medium was added. After a further 48 h incubation, cells were harvested by centrifuging at 500 g for 10 min. Supernatant containing viral particles was filtered through a 0.45-μm filter and the lentivirus titer determined by enzyme-linked immunosorbent assay (ELISA) with the Lenti-X p24 Rapid Titer (Single Wash) Kit (Takara). To generate cell lines expressing MDA5 variants, A549 RIG-I^−/−^ ACE2^+^ cells (80–90% confluent in 6-well plates) were transduced by adding 10 μg mL^−1^ Polybrene (Tocris) and recombinant lentivirus to a multiplicity of infection (MOI) between 2 and 10, followed by centrifugation at 800*g* for 30 min. After 16 h the transduction medium was exchanged for fresh growth medium. After a further 48 h incubation, 2 μg mL^−1^ puromycin was added and antibiotic selection maintained for two weeks. MDA5 expression was assessed in the presence of 0–100 μg mL^−1^ doxycycline by Western blotting with anti-FLAG (Sigma-Aldrich, RRID: AB_262044, 1:5000 dilution) or anti-MDA5 antibody (Enzo Life Sciences, RRID:AB_2893162, 1:1000 dilution).

#### Cell culture and induction

A549 (RIG-I^−/−^ ACE2^+^) cells expressing MDA5 variants were grown in high glucose DMEM, supplemented with 10% FBS. Following overnight recovery in DMEM (10% FBS), 2.5 x 10^5^ A549 cells were seeded into 6-well plates containing DMEM (10% FBS). To activate MDA5 expression, cells were supplemented with 1 μg mL^−1^ doxycycline. After 24 h incubation, cells were lysed to assess expression levels of endogenous and knocked-in MDA5. For poly(I:C) induction, 1.0 x 10^6^ A549 cells were seeded into 6-well plates and incubated for 24 h before transfection with 1250 ng high-molecular weight poly(I:C) (InvivoGen, cat. no. tlrl-pic) per well, using 3.5 μL Lipofectamine MessengerMAX Transfection Reagent (Invitrogen) in Opti-MEM Medium (Gibco). Cells were incubated for an additional 24 h post-transfection before lysis.

#### Immunoblotting to measure MDA5 expression

Cells were washed twice with TBS, supplemented with 0.05% tween (TBS-T). Cells were lysed in RIPA buffer (50mM Tris-HCL (pH 7.4), 50 mM NaCl, 2 mM EDTA, 0.1% SDS), supplemented with cOmplete EDTA-free Protease Inhibitor Cocktail (Roche, cat. no. 11873580001) and PhosSTOP (Roche, cat. no. 04906845001). Cells were agitated on a rotator for 30 min at 4°C, and cell debris pelleted at 16,000 g for 20 min at 4°C. Clarified lysates were heated to 95°C and separated on NuPAGE 4–12% Bis-Tris gels. Gels were blotted onto a nitrocellulose membrane using the iBlot system (Invitrogen), and membranes blocked in 5% milk in TBS-T. MDA5 expression was assessed by Western blotting using an anti-MDA5 antibody (Enzo Life Sciences, RRID:AB_2893162, 1:1000 dilution) and anti-β actin antibody (Antibodies.com, RRID:AB_2748874, 1:1000 dilution) as a loading control.

#### Virus infection assays

Recombinant encephalomyocarditis virus (EMCV, strain Mengovirus) Leader-Zn mutant (carrying mutations C19A and C22A in the zinc finger domain of the viral Leader protein)^55^ was generated by transfecting RNA produced from an infectious clone into BHK-21 cells. Viruses were harvested after complete cytopathogenic effect, concentrated by ultracentrifugation (30% sucrose, 140,000 g for 16 h, 4°C, SW32Ti rotor), diluted in PBS and stored at −80°C. Virus titers were determined by endpoint titration according to the method of Spearman-Kärber and expressed as 50% Tissue Culture Infectious dose (TCID50). EMCV was used to infect A549 RIG-I^−/−^ ACE2^+^ cells expressing MDA5 variants cultured in high glucose DMEM, supplemented with 1 μg mL^−1^ doxycycline, 10% FBS, and 1% Pen-Strep. Concentrations for *IFNB1* mRNA, *IFIT1* mRNA, and EMCV viral RNA (vRNA) were quantified by RT-qPCR from total RNA extracted from the infected cells 7 h post infection as described previously (Figure S1).^65^

#### MDA5 protein purification

A gene encoding mouse MDA5 (Ifih1, UniProt: Q8R5F7) was cloned into the pET28a vector with an N-terminal hexahistidine tag followed by a tobacco etch virus (TEV) protease cleavage site as described.^19^ MDA5 residues 646–663, in the flexible L2 surface loop of the helicase 2 insert domain (Hel2i), were deleted for solubility, resulting in a 114-kDa polypeptide chain. The ΔL2 loop deletion does not affect the dsRNA binding, ATPase or interferon signaling activities of MDA5.^19,21,22^ The T1D-protective mutations were introduced into the MDA5-ΔL2 construct with the Q5 Site-Directed Mutagenesis Kit (New England BioLabs).

*E. coli* Rosetta(DE3)pLysS cells (Novagen, cat. no. 71403) were transformed with an MDA5 construct and grown in 2xTY medium to OD_600_ 0.4–0.6 at 37°C. After cooling to 16°C, protein expression was induced with 0.5 mM isopropyl-β-D-1-thiogalactopyranoside (IPTG) overnight at 16°C. Harvested cells were resuspended in lysis buffer (30 mM HEPES pH 7.7, 500 mM M NaCl, 5mM MgCl_2_, 5% glycerol, 1 mM Tris(2-carboxyethyl)phosphine (TCEP)), with cOmplete EDTA-free Protease Inhibitor Cocktail (Roche, cat. no. 11873580001) and 1 U ml^−1^ Salt Active Nuclease (Merck, cat. no. SRE0015). Cells were lysed by sonication (5s s on, 10 s off, 40% power), and the lysate was centrifuged at 37,500 g for 1 h. The supernatant was loaded onto a pre-equilibrated 5 Ml HisTrap HP column (Cytiva, cat. no. 17-5248-02), washed with wash buffer (30 mM HEPES pH 7.7, 500 mM NaCl, 20 mM imidazole, 5% glycerol, and 1 mM TCEP). MDA5 was eluted with Ni-NTA elution buffer (30 mM HEPES 7.7, 300 mM NaCl, 250 mM imidazole, 5% glycerol, 1 mM TCEP). MDA5 was further purified on a Resource Q anion exchange column (Cytiva) (buffer A: 20 mM HEPES 7.7, 50 mM NaCl, 1 mM dithiothreitol (DTT); buffer B: 20 mM HEPES 7.7, 1 M NaCl, 1 mM DTT), and a Superdex 200 Increase 10/300 GL size-exclusion column (Cytiva) in SEC buffer (20 mM HEPES pH 7.7, 150 mM M KCl, 1 mM DTT). Purified protein was used immediately for cryo-EM grid preparation and ATPase assays, or flash-frozen (with 5% glycerol) and stored at −80°C.

#### Nanoscale differential scanning fluorimetry (nanoDSF)

1 μM MDA5 in buffer (20 mM HEPES pH 7.7, 150 mM KCl, 1 mM DTT), alone or incubated with 15 ng μL^−1^ (22 nM) 1-kb dsRNA for 30 min at room temperature, was loaded into standard capillaries (NanoTemper, #PR-C002). Intrinsic protein fluorescence at 330 nm and 350 nm, F330 and F350, respectively, was measured from 15°C to 80°C, with a heating rate of 1°C per minute, with a Prometheus NT.48 nano-fluorimeter (NanoTemper). The melting temperatures (T_m_ values) were calculated from changes in the fluorescence ration (F350/F330) using the PR.Stability Analysis v1.1 software (NanoTemper).

#### Bio-layer interferometry (BLI)

BLI experiments were performed on an Octet Red384 (ForteBio Inc.) instrument. Binding experiments were carried out at 30°C in assay buffer (20 mM HEPES pH 7.7, 150 mM KCl, 1 mM DTT, 2 mg mL^−1^ BSA). BSA was required in the assay buffer to avoid non-specific binding to the sensor.

Pierce RNA 3′ End Biotinylation Kit (ThermoFisher, cat. no. 20160) was used to label the 3′ end of 200-bp, 300-bp or 1-kb RNA duplexes (produced as described above) with biotin. Biotinylated RNA (2.5 μg mL^−1^) was immobilized onto Octet streptavidin (SA) Biosensors (Sartorius), and binding performed for varying concentrations of MDA5 (Figure S2). Sensors were hydrated in assay buffer for at least 600 s prior to all measurements. Binding experiments comprised sensor equilibration (60 s), loading (600 s), baseline (90 s), and association and dissociation (600 s each) steps. To determine the effect of ATP on MDA5 dissociation rates, 4 mM ATP was added to assay buffer in the dissociation step, and dissociation rate constants (*k*_off_) calculated from the first 60 s of the dissociation step.

Data analysis was performed using the Octet Analysis Studio v 11.1 software (Sartorius). Loaded sensors dipped into assay buffer during the association and dissociation steps were used as references and subtracted from all samples during analysis to correct for baseline drift. Local kinetic fitting was used to determine K_d_ values from binding curves with 125 nM MDA5. K_d_ values obtained from three or four replicate runs were averaged, and standard error calculated, using GraphPad Prism v10.

#### ATPase assay

ATPase activities were measured using the ATPase/GTPase Activity Assay Kit (Sigma-Aldrich, cat. no. MAK113). Reactions containing 180 nM MDA5 and 25 ng μL^−1^ 1-kb RNA in buffer (20 mM HEPES pH 7.7, 150 mM KCl, 4 mM ATP, 4 mM MgSO_4_, 1 mM DTT) were incubated at 37°C for 15 min and quenched by the addition of malachite green. Reactions were performed in clear, flat-bottom 96-well plates, and the inorganic phosphate produced by ATP hydrolysis was monitored by tracking absorbance at 620 nm using a CLARIOstar microplate reader (BMG Labtech). Results were analyzed with Prism v10 (GraphPad), and for mutants, data was normalized relative to the phosphate generated by wild type MDA5.

#### Negative stain EM

200 nM MDA5 and 3 ng μL^−1^ (4.4 nM) 1-kb dsRNA were incubated at room temperature for 30 min in 20 mM HEPES pH 7.7, 150 mM KCl, 1 mM DTT. Carbon film 300-mesh grids (Agar Scientific) were glow discharged at 25 mA for 1 min. Samples were applied to the grids, washed with RNase-free water, stained using uranyl acetate [2% (w/v)], and imaged with a 120 kV Technai G2 Spirit electron microscope (ThermoFisher). Images were taken at −2 to −4 μm defocus and 15,000× magnification (3.5 Å pixel^−1^). Filament lengths were quantified with Fiji v2.16^66^.

#### Cryo-EM sample preparation and data collection

For data collection of MDA5 bound to ATP or ADP-AlF_4_, 1 g L^−1^ purified MDA5 protein was incubated on ice for 2–3 min with 0.05 g L^−1^ 1-kb dsRNA in 20 mM HEPES pH 7.7, 100 mM KCl, 5 mM MgCl_2_, 2 mM DTT, and either 10 mM ATP or 2 mM ADP, 4 mM AlCl_3_ and 40 mM NaF. Samples were diluted 2-fold with the same buffer and 3.5 μL of sample was immediately applied onto a glow-discharged 300-mesh gold Quantifoil R1.2/1.3 grid (Quantifoil Micro Tools). Grids were glow discharged with an Edwards 12E6/531 glow discharger at 30 mA for 60 s. Grids were blotted for 2–4 s, held for a 15 s wait time, and plunge-frozen in liquid ethane cooled by liquid nitrogen with a Vitrobot Mark IV (ThermoFisher) at 4°C and 100% humidity.

Cryo-EM data were collected on 300 kV Titan Krios microscopes (ThermoFisher) equipped with Gatan K3 detectors and Gatan BioQuantum energy filters at the MRC Laboratory of Molecular Biology and EMBL Heidelberg. Movies were recorded with a fluence of 40–48 electrons per square angstrom (e^−^ Å^−2^), an average exposure of 1.0 e^−^ Å^−2^ per frame, and a flux of 5–5.7 e^−^ pixel^−1^ s^−1^. A 20-eV energy selection slit width was used. The nominal defocus value ranged from −0.5 to −2.5 μm in 0.5 μm increments. All I923V ATP samples were collected on a K3 detector (Gatan) at 105,000x magnification (0.826 Å pixel^−1^). The A946T sample was collected at 96,000x magnification (0.822 Å pixel^−1^). Detectors were used in counting mode. Data were acquired with EPU (ThermoFisher) and two shots per hole, except for the A946T sample, which was acquired with SerialEM^67^ and six shots per hole. See Table S1 for additional data collection parameters.

#### Image processing and helical reconstruction

Movies were motion-corrected and dose-weighted with MotionCor2.0 in Relion4.0.^68^ The contrast transfer function was estimated with CtfFind4.1^69^ and the micrographs were aligned without dose weighting. Image reconstruction with helical symmetry averaging was performed in Relion4.0.^70^ Segments were picked with crYOLO^71^ from a template trained on the M854K ATP dataset (EMD-12213).^23^ Segment were subjected to several rounds of 2D and 3D classification. For datasets collected in the presence of nucleotide, all 3D classes contained density for the nucleotide. 3D classes with the highest overall resolution were selected for 3D refinement, CTF refinement, Bayesian polishing, and post-processing, which were performed in Relion4.0.^68,72^ To calculate the helical twist distribution of helical segments, three independent rounds of 3D classification were performed with five classes. Segments were then placed in Low twist (71°–81°), Intermediate twist (81°–91°) or High twist (91°–96°) bins for plotting histograms of the twist distribution with Prism v10 (GraphPad). The number of segments contributing to each 3D class was weighted evenly. The numbers of segments used in 3D classification for twist distribution calculation datasets were as follows: I923V ATP dataset, 457,811 segments; I923V no-nucleotide dataset, 547,562 segments; and I923V ADP-AlF_4_ dataset, 683,412 segments. See Table S1 for the initial and final number of segments used for each dataset and for the final resolution and helical parameters of each reconstruction (Figure S3).

#### Model building and refinement

Previously determined cryo-EM structures of MDA5-dsRNA filaments with similar helical symmetry (PDB:7BKP, [https://doi.org/10.2210/pdb7BKP/pdb], PDB:7NIC, [https://doi.org/10.2210/pdb7NIC/pdb], or PDB:7NIQ, [https://doi.org/10.2210/pdb7NIQ/pdb])^22^ were used as the starting atomic models for model building. The model was docked into the density for the central subunit in each map with the Fit in Map function of UCSF Chimera.^73^ The docked models were rebuilt with COOT.^74^ Models of adjacent protomers were generated in Chimera by applying the helical symmetry calculated in Relion. The resulting models with three MDA5 subunits were used for subsequent real space refinement in Phenix 1.21.^75^ Real space refinement in Phenix included global minimization and atomic displacement parameter refinement, incorporating restraints on secondary structure, sidechain rotamers, mainchain torsion angles, and non-crystallographic symmetry between the three modeled protein subunits.^75^

To determine which conformational state the helicase modules were in, each model was superimposed onto the fully closed structure of LGP2 (PDB:5JAJ)^9^ using the secondary structure elements of Hel1 as the reference. The conformational state of the helicase domain was defined based on the rotation angles relating the Hel2 domains of the aligned structures as follows: closed state, <5° angle; semi-closed state, >5° angle. Protein interfaces were analyzed with the Protein interfaces, surfaces, and assemblies (PISA) server at the European Bioinformatics Institute [http://www.ebi.ac.uk/pdbe/prot_int/pistart.html].^61^

### Quantification and Statistical Analysis

No statistical methods were used to predetermine sample size, experiments were not randomized, and the investigators were not blinded to experimental outcomes. Cell signaling data and BLI data are represented as the mean ± standard error of the mean of at least three replicates conducted in three independent experiments. ATPase assays were performed at least three times in independent experiments. Scatterplots, histograms and error bars were plotted with GraphPad Prism v.10.2.3 and Microsoft Excel v.16.84. Statistical significance was assessed using unpaired two-tailed t-tests assuming Gaussian distributions (without Welch’s correction). Statistical significance was assigned as follows: n.s., *p* > 0.05; *, *p* < 0.05; **, *p* < 0.01; ***, *p* < 0.001.

## Supplementary Material

**Supplemental Information**

Supplemental information can be found online at https://doi.org/10.1016/j.celrep.2025.115754.

Supplementary Material

## Data Availability

The atomic coordinates were deposited in the Protein Data Bank with accession codes PDB: 9F2W at https://doi.org/10.2210/pdb9f2w/pdb, PDB: 9F2L at https://doi.org/10.2210/pdb9f2l/pdb, PDB: 9F1U at https://doi.org/10.2210/pdb9F1U/pdb, PDB: 9F20 at https://doi.org/10.2210/pdb9f20/pdb, PDB: 9F0J at https://doi.org/10.2210/pdb9f0j/pdb, and PDB: 9F3P at https://doi.org/10.2210/pdb9f3p/pdb. The cryo-EM densities were deposited in the Electron Microscopy Data Bank with codes EMD-50165, EMD-50150, EMD-50136, EMD-50137, EMD-50111, and EMD-50175. No original code was generated as part of this study. Other data underlying this article are available in the article, Figures S1–S4, Table S1, and Data S1.

## Highlights

- MDA5 genetic variants can protect from autoimmune disease via multiple mechanisms
- Rare variants E627* and I923V inhibit cellular IFN induction in picornavirus infection
- E627* does not bind RNA; I923V is ATPase hyperactive, causing premature dissociation from RNA
- R843H/T946A do not affect cytokine induction, suggesting an indirect disease mechanism

## References

[1] Hur S (2019). Double-Stranded RNA Sensors and Modulators in Innate Immunity. Annu Rev Immunol.

[2] Kato H, Sato S, Yoneyama M, Yamamoto M, Uematsu S, Matsui K, Tsujimura T, Takeda K, Fujita T, Takeuchi O, Akira S (2005). Cell type-specific involvement of RIG-I in antiviral response. Immunity.

[3] Yoneyama M, Kikuchi M, Matsumoto K, Imaizumi T, Miyagishi M, Taira K, Foy E, Loo YM, Gale M, Akira S (2005). Shared and unique functions of the DExD/H-box helicases RIG-I, MDA5, and LGP2 in antiviral innate immunity. J Immunol.

[4] Kato H, Takeuchi O, Sato S, Yoneyama M, Yamamoto M, Matsui K, Uematsu S, Jung A, Kawai T, Ishii KJ (2006). Differential roles of MDA5 and RIG-I helicases in the recognition of RNA viruses. Nature.

[5] Kato H, Takeuchi O, Mikamo-Satoh E, Hirai R, Kawai T, Matsushita K, Hiiragi A, Dermody TS, Fujita T, Akira S (2008). Length-dependent recognition of double-stranded ribonucleic acids by retinoic acid-inducible gene-I and melanoma differentiation-associated gene 5. J Exp Med.

[6] Venkataraman T, Valdes M, Elsby R, Kakuta S, Caceres G, Saijo S, Iwakura Y, Barber GN (2007). Loss of DExD/H box RNA helicase LGP2 manifests disparate antiviral responses. J Immunol.

[7] Pippig DA, Hellmuth JC, Cui S, Kirchhofer A, Lammens K, Lammens A, Schmidt A, Rothenfusser S, Hopfner KP (2009). The regulatory domain of the RIG-I family ATPase LGP2 senses double-stranded RNA. Nucleic Acids Res.

[8] Li X, Ranjith-Kumar CT, Brooks MT, Dharmaiah S, Herr AB, Kao C, Li P (2009). The RIG-I-like receptor LGP2 recognizes the termini of double-stranded RNA. J Biol Chem.

[9] Uchikawa E, Lethier M, Malet H, Brunel J, Gerlier D, Cusack S (2016). Structural Analysis of dsRNA Binding to Anti-viral Pattern Recognition Receptors LGP2 and MDA5. Mol Cell.

[10] Satoh T, Kato H, Kumagai Y, Yoneyama M, Sato S, Matsushita K, Tsujimura T, Fujita T, Akira S, Takeuchi O (2010). LGP2 is a positive regulator of RIG-I- and MDA5-mediated antiviral responses. Proc Natl Acad Sci USA.

[11] Kumar A, Haque J, Lacoste J, Hiscott J, Williams BR (1994). Double-stranded RNA-dependent protein kinase activates transcription factor NF-kappa B by phosphorylating I kappa B. Proc Natl Acad Sci USA.

[12] Vijay-Kumar M, Gentsch JR, Kaiser WJ, Borregaard N, Offermann MK, Neish AS, Gewirtz AT (2005). Protein kinase R mediates intestinal epithelial gene remodeling in response to double-stranded RNA and live rotavirus. J Immunol.

[13] Chebath J, Benech P, Revel M, Vigneron M (1987). Constitutive expression of (2’-5’) oligo A synthetase confers resistance to picornavirus infection. Nature.

[14] Kristiansen H, Scherer CA, McVean M, Iadonato SP, Vends S, Thavachelvam K, Steffensen TB, Horan KA, Kuri T, Weber F (2010). Extracellular 2’-5’ oligoadenylate synthetase stimulates RNase L-independent antiviral activity: a novel mechanism of virus-induced innate immunity. J Virol.

[15] Bauernfried S, Scherr MJ, Pichlmair A, Duderstadt KE, Hornung V (2021). Human NLRP1 is a sensor for double-stranded RNA. Science.

[16] Yin X, Riva L, Pu Y, Martin-Sancho L, Kanamune J, Yamamoto Y, Sakai K, Gotoh S, Miorin L, De Jesus PD (2021). MDA5 Governs the Innate Immune Response to SARS-CoV-2 in Lung Epithelial Cells. Cell Rep.

[17] Rebendenne A, Valadao ALC, Tauziet M, Maarifi G, Bonaventure B, McKellar J, Planes R, Nisole S, Arnaud-Arnould M, Moncorge O, Goujon C (2021). SARS-CoV-2 triggers an MDA-5-dependent interferon response which is unable to control replication in lung epithelial cells. J Virol.

[18] Peisley A, Lin C, Wu B, Orme-Johnson M, Liu M, Walz T, Hur S (2011). Cooperative assembly and dynamic disassembly of MDA5 filaments for viral dsRNA recognition. Proc Natl Acad Sci USA.

[19] Berke IC, Modis Y (2012). MDA5 cooperatively forms dimers and ATP-sensitive filaments upon binding double-stranded RNA. EMBO J.

[20] Berke IC, Yu X, Modis Y, Egelman EH (2012). MDA5 assembles into a polar helical filament on double-stranded RNA. Proc Natl Acad Sci USA.

[21] Wu B, Peisley A, Richards C, Yao H, Zeng X, Lin C, Chu F, Walz T, Hur S (2013). Structural basis for dsRNA recognition, filament formation, and antiviral signal activation by MDA5. Cell.

[22] Yu Q, Qu K, Modis Y (2018). Cryo-EM Structures of MDA5-dsRNA Filaments at Different Stages of ATP Hydrolysis. Mol Cell.

[23] Yu Q, Herrero Del Valle A, Singh R, Modis Y (2021). MDA5 disease variant M854K prevents ATP-dependent structural discrimination of viral and cellular RNA. Nat Commun.

[24] Hou F, Sun L, Zheng H, Skaug B, Jiang QX, Chen ZJ (2011). MAVS forms functional prion-like aggregates to activate and propagate antiviral innate immune response. Cell.

[25] Wu B, Peisley A, Tetrault D, Li Z, Egelman EH, Magor KE, Walz T, Penczek PA, Hur S (2014). Molecular imprinting as a signal-activation mechanism of the viral RNA sensor RIG-I. Mol Cell.

[26] Kagan JC, Magupalli VG, Wu H (2014). SMOCs: supramolecular organizing centres that control innate immunity. Nat Rev Immunol.

[27] Ahmad S, Mu X, Yang F, Greenwald E, Park JW, Jacob E, Zhang CZ, Hur S (2018). Breaching Self-Tolerance to Alu Duplex RNA Underlies MDA5-Mediated Inflammation. Cell.

[28] Chung H, Calis JJA, Wu X, Sun T, Yu Y, Sarbanes SL, Thi Dao, Shilvock AR, Hoffmann HH, Rosenberg BR, Rice CM (2018). Human ADAR1 Prevents Endogenous RNA from Triggering Translational Shutdown. Cell.

[29] Liddicoat BJ, Piskol R, Chalk AM, Ramaswami G, Higuchi M, Hartner JC, Li JB, Seeburg PH, Walkley CR (2015). RNA editing by ADAR1 prevents MDA5 sensing of endogenous dsRNA as nonself. Science.

[30] Singh R, Wu Y, Herrero Del Valle A, Leigh KE, Mong S, Cheng MTK, Ferguson BJ, Modis Y (2024). Contrasting functions of ATP hydrolysis by MDA5 and LGP2 in viral RNA sensing. J Biol Chem.

[31] Rodero MP, Crow YJ (2016). Type I interferon-mediated monogenic autoinflammation: The type I interferonopathies, a conceptual overview. J Exp Med.

[32] Rutsch F, MacDougall M, Lu C, Buers I, Mamaeva O, Nitschke Y, Rice GI, Erlandsen H, Kehl HG, Thiele H (2015). A specific IFIH1 gain-of-function mutation causes Singleton-Merten syndrome. Am J Hum Genet.

[33] Rice GI, Del Toro Duany Y, Jenkinson EM, Forte GM, Anderson BH, Ariaudo G, Bader-Meunier B, Baildam EM, Battini R, Beresford MW (2014). Gain-of-function mutations in IFIH1 cause a spectrum of human disease phenotypes associated with upregulated type I interferon signaling. Nat Genet.

[34] Rice GI, Park S, Gavazzi F, Adang LA, Ayuk LA, Van Eyck L, Seabra L, Barrea C, Battini R, Belot A (2020). Genetic and phenotypic spectrum associated with IFIH1 gain-of-function. Hum Mutat.

[35] Takeichi T, Katayama C, Tanaka T, Okuno Y, Murakami N, Kono M, Sugiura K, Aoyama Y, Akiyama M (2018). A novel IFIH1 mutation in the pincer domain underlies the clinical features of both Aicardi-Goutieres and Singleton-Merten syndromes in a single patient. Br J Dermatol.

[36] Garau J, Cavallera V, Valente M, Tonduti D, Sproviero D, Zucca S, Battaglia D, Battini R, Bertini E, Cappanera S (2019). Molecular Genetics and Interferon Signature in the Italian Aicardi Goutieres Syndrome Cohort: Report of 12 New Cases and Literature Review. J Clin Med.

[37] Smyth DJ, Cooper JD, Bailey R, Field S, Burren O, Smink LJ, Guja C, Ionescu-Tirgoviste C, Widmer B, Dunger DB (2006). A genome-wide association study of nonsynonymous SNPs identifies a type 1 diabetes locus in the interferon-induced helicase (IFIH1) region. Nat Genet.

[38] Nejentsev S, Walker N, Riches D, Egholm M, Todd JA (2009). Rare variants of IFIH1, a gene implicated in antiviral responses, protect against type 1 diabetes. Science.

[39] Liu S, Wang H, Jin Y, Podolsky R, Reddy MVPL, Pedersen J, Bode B, Reed J, Steed D, Anderson S (2009). IFIH1 polymorphisms are significantly associated with type 1 diabetes and IFIH1 gene expression in peripheral blood mononuclear cells. Hum Mol Genet.

[40] Vasseur E, Patin E, Laval G, Pajon S, Fornarino S, Crouau-Roy B, Quintana-Murci L (2011). The selective footprints of viral pressures at the human RIG-I-like receptor family. Hum Mol Genet.

[41] de Azevedo Silva J, Tavares NA, Santos MM, Moura R, Guimaraes RL, Araujo J, Crovella S, Brandao LA (2015). Meta-analysis of STAT4 and IFIH1 polymorphisms in type 1 diabetes mellitus patients with autoimmune polyglandular syndrome type III. Genet Mol Res.

[42] Gorman JA, Hundhausen C, Errett JS, Stone AE, Allenspach EJ, Ge Y, Arkatkar T, Clough C, Dai X, Khim S (2017). The A946T variant of the RNA sensor IFIH1 mediates an interferon program that limits viral infection but increases the risk for autoimmunity. Nat Immunol.

[43] Jermendy Á, Szatmári I, Körner A, Szabó AJ, Tóth-Heyn P, Hermann R (2018). Association between interferon-induced helicase (IFIH1) rs1990760 polymorphism and seasonal variation in the onset of type 1 diabetes mellitus. Pediatr Diabetes.

[44] Pedergnana V, Abdel-Hamid M, Guergnon J, Theodorou I, Fontanet A, Abel L, Cobat A (2016). Refined association of melanoma differentiation-associated gene 5 variants with spontaneous hepatitis C virus clearance in Egypt. Hepatology.

[45] Vergara C, Thio CL, Thomas D, Duggal P (2016). Polymorphisms in melanoma differentiation-associated gene 5 are not associated with clearance of hepatitis C virus in a European American population. Hepatology.

[46] Shigemoto T, Kageyama M, Hirai R, Zheng J, Yoneyama M, Fujita T (2009). Identification of loss of function mutations in human genes encoding RIG-I and MDA5: implications for resistance to type I diabetes. J Biol Chem.

[47] Hoffmann FS, Schmidt A, Dittmann Chevillotte M, Wisskirchen C, Hellmuth J, Willms S, Gilmore RH, Glas J, Folwaczny M, Müller T (2015). Polymorphisms in MDA-5 link protein function to clearance of hepatitis C virus. Hepatology.

[48] Lincez PJ, Shanina I, Horwitz MS (2015). Reduced expression of the MDA5 Gene IFIH1 prevents autoimmune diabetes. Diabetes.

[49] Zouk H, Marchand L, Li Q, Polychronakos C (2014). Functional characterization of the Thr946Ala SNP at the type 1 diabetes IFIH1 locus. Autoimmunity.

[50] Marinou I, Montgomery DS, Dickson MC, Binks MH, Moore DJ, Bax DE, Wilson AG (2007). The interferon induced with helicase domain 1 A946T polymorphism is not associated with rheumatoid arthritis. Arthritis Res Ther.

[51] Downes K, Pekalski M, Angus KL, Hardy M, Nutland S, Smyth DJ, Walker NM, Wallace C, Todd JA (2010). Reduced expression of IFIH1 is protective for type 1 diabetes. PLoS One.

[52] Hyoty H, Taylor KW (2002). The role of viruses in human diabetes. Diabetologia.

[53] Wang JP, Cerny A, Asher DR, Kurt-Jones EA, Bronson RT, Finberg RW (2010). MDA5 and MAVS mediate type I interferon responses to coxsackie B virus. J Virol.

[54] Visser LJ, Langereis MA, Rabouw HH, Wahedi M, Muntjewerff EM, de Groot RJ, van Kuppeveld FJM (2019). Essential Role of Enterovirus 2A Protease in Counteracting Stress Granule Formation and the Induction of Type I Interferon. J Virol.

[55] Hato SV, Ricour C, Schulte BM, Lanke KHW, de Bruijni M, Zoll J, Melchers WJG, Michiels T, van Kuppeveld FJM (2007). The mengovirus leader protein blocks interferon-alpha/beta gene transcription and inhibits activation of interferon regulatory factor 3. Cell Microbiol.

[56] Dou Y, Yim HC, Kirkwood CD, Williams BR, Sadler AJ (2017). The innate immune receptor MDA5 limits rotavirus infection but promotes cell death and pancreatic inflammation. EMBO J.

[57] Lincez PJ, Shanina I, Horwitz MS (2021). Changes in MDA5 and TLR3 Sensing of the Same Diabetogenic Virus Result in Different Autoimmune Disease Outcomes. Front Immunol.

[58] Domsgen E, Lind K, Kong L, Hühn MH, Rasool O, van Kuppeveld F, Korsgren O, Lahesmaa R, Flodström-Tullberg M (2016). An IFIH1 gene polymorphism associated with risk for autoimmunity regulates canonical antiviral defence pathways in Coxsackievirus infected human pancreatic islets. Sci Rep.

[59] Teague HC, Lefevre C, Rieser E, Wolfram L, de Miguel D, de Oliveira Patricio, Oliveira M, Mansur DS, Irigoyen N, Walczak H, Ferguson BJ (2024). LUBAC is required for RIG-I sensing of RNA viruses. Cell Death Differ.

[60] Funabiki M, Kato H, Miyachi Y, Toki H, Motegi H, Inoue M, Minowa O, Yoshida A, Deguchi K, Sato H (2014). Autoimmune disorders associated with gain of function of the intracellular sensor MDA5. Immunity.

[61] Krissinel E, Henrick K (2007). Inference of macromolecular assemblies from crystalline state. J Mol Biol.

[62] Wallace C, Singh R, Modis Y (2025). MDA5 variants trade antiviral activity for protection from autoimmune disease. medRxiv.

[63] Yeung WCG, Rawlinson WD, Craig ME (2011). Enterovirus infection and type 1 diabetes mellitus: systematic review and meta-analysis of observational molecular studies. BMJ.

[64] Axelrad JE, Olen O, Askling J, Lebwohl B, Khalili H, Sachs MC, Ludvigsson JF (2019). Gastrointestinal Infection Increases Odds of Inflammatory Bowel Disease in a Nationwide Case-Control Study. Clin Gastroenterol Hepatol.

[65] Bruurs LJM, Müller M, Schipper JG, Rabouw HH, Boersma S, van Kuppeveld FJM, Tanenbaum ME (2023). Antiviral responses are shaped by heterogeneity in viral replication dynamics. Nat Microbiol.

[66] Schindelin J, Arganda-Carreras I, Frise E, Kaynig V, Longair M, Pietzsch T, Preibisch S, Rueden C, Saalfeld S, Schmid B (2012). Fiji: an open-source platform for biological-image analysis. Nat Methods.

[67] Mastronarde DN (2005). Automated electron microscope tomography using robust prediction of specimen movements. J Struct Biol.

[68] Kimanius D, Dong L, Sharov G, Nakane T, Scheres SHW (2021). New tools for automated cryo-EM single-particle analysis in RELION-4.0. Biochem J.

[69] Rohou A, Grigorieff N (2015). CTFFIND4: Fast and accurate defocus estimation from electron micrographs. J Struct Biol.

[70] He S, Scheres SHW (2017). Helical reconstruction in RELION. J Struct Biol.

[71] Wagner T, Merino F, Stabrin M, Moriya T, Antoni C, Apelbaum A, Hagel P, Sitsel O, Raisch T, Prumbaum D (2019). SPHIRE-crY-OLO is a fast and accurate fully automated particle picker for cryo-EM. Commun Biol.

[72] Scheres SH (2014). Beam-induced motion correction for sub-megadalton cryo-EM particles. Elife.

[73] Pettersen EF, Goddard TD, Huang CC, Couch GS, Greenblatt DM, Meng EC, Ferrin TE (2004). UCSF Chimera–a visualization system for exploratory research and analysis. J Comput Chem.

[74] Emsley P, Cowtan K (2004). Coot: model-building tools for molecular graphics. Acta Crystallogr D Biol Crystallogr.

[75] Liebschner D, Afonine PV, Baker ML, Bunkóczi G, Chen VB, Croll TI, Hintze B, Hung LW, Jain S, McCoy AJ (2019). Macromolecular structure determination using X-rays, neutrons and electrons: recent developments in Phenix. Acta Crystallogr D Struct Biol.

