# Supplementary material for: Molecular basis of autoimmune disease protection by MDA5 variants
Source: Cell Rep. Author manuscript; Available in PMC 2025 Nov 18. (PMC7618376; doi:10.1016/j.celrep.2025.115754)
Supplement: Supplementary Material [file EMS210629-supplement-Supplementary_Material.zip › 1-s2.0-S221112472500525X-mmc1.pdf]

Cell Reports, Volume *44*

Supplemental information

Molecular basis of autoimmune

disease protection by MDA5 variants

Rahul Singh, Joe D. Joiner, Alba Herrero del Valle, Marleen Zwaagstra, Ida Jobe, Brian J. Ferguson, Frank J.M. van Kuppeveld, and Yorgo Modis

Supplementary Figures and Table

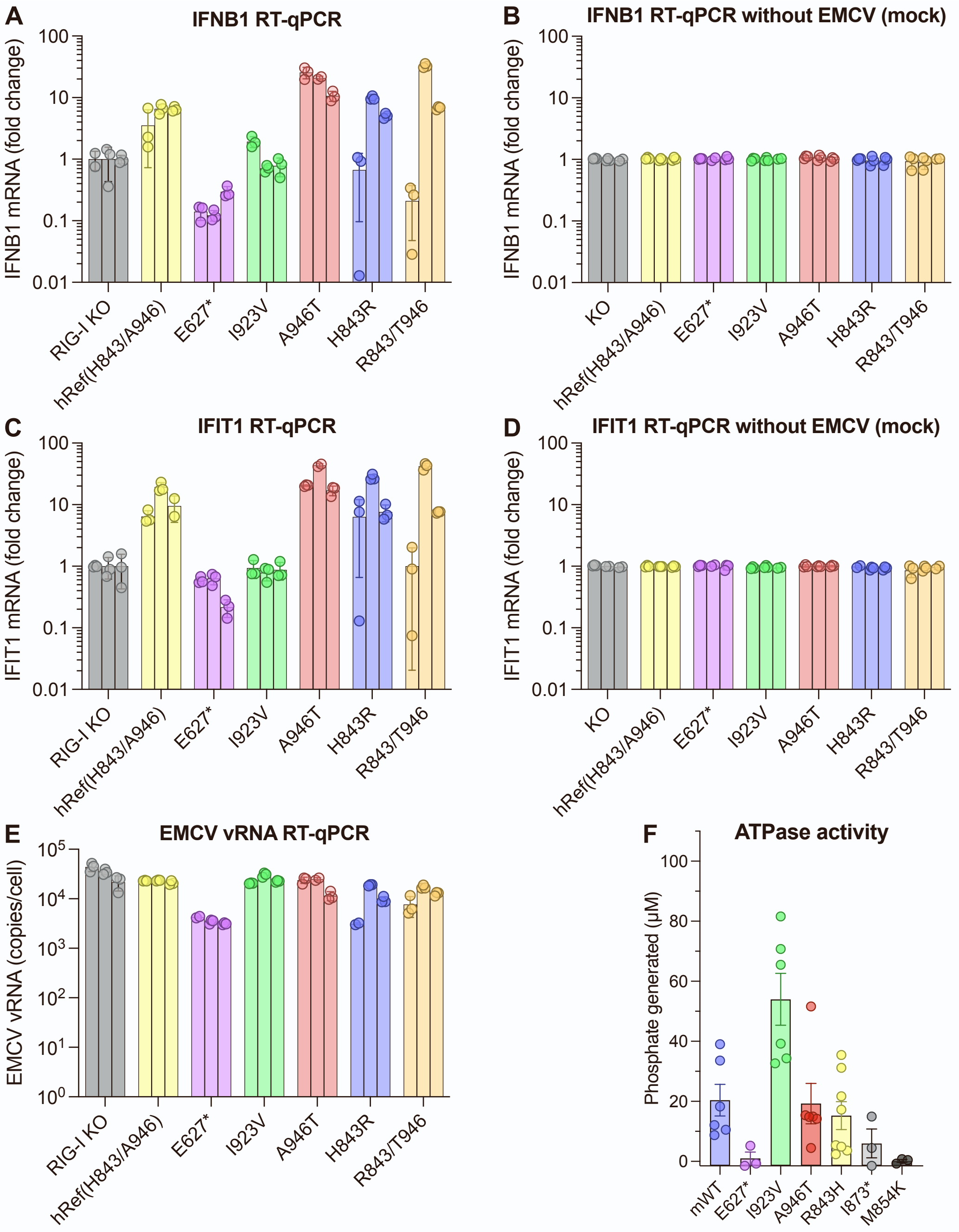

Fig. S1. Encephalomyocarditis virus (EMCV) data with technical replicates shown and EMCV mock infections (related to Figure 1). (**A,B**) RT-qPCR quantification of IFNB1 mRNA in A549 RIG-I KO cells stably expressing the indicated MDA5 variant under a doxycycline-inducible promoter 7 h after infection with EMCV, (**A**), or 7 h after addition of mock buffer, (**B**). hRef, human reference sequence. (**C,D**) RT-qPCR quantification of IFIT1 mRNA after EMCV infection, (**C**), or without EMCV infection, (**D**). (**E**) RT-qPCR quantification of EMCV RNA. All technical replicates from 3 independent experiments are shown. (**F**) ATPase activities of mouse MDA5 variants expressed as concentration of phosphate generated. Error bars represent mean ± SEM (3 or 6 measurements from 1 or 2 independent experiments). See Data S1 for source data.

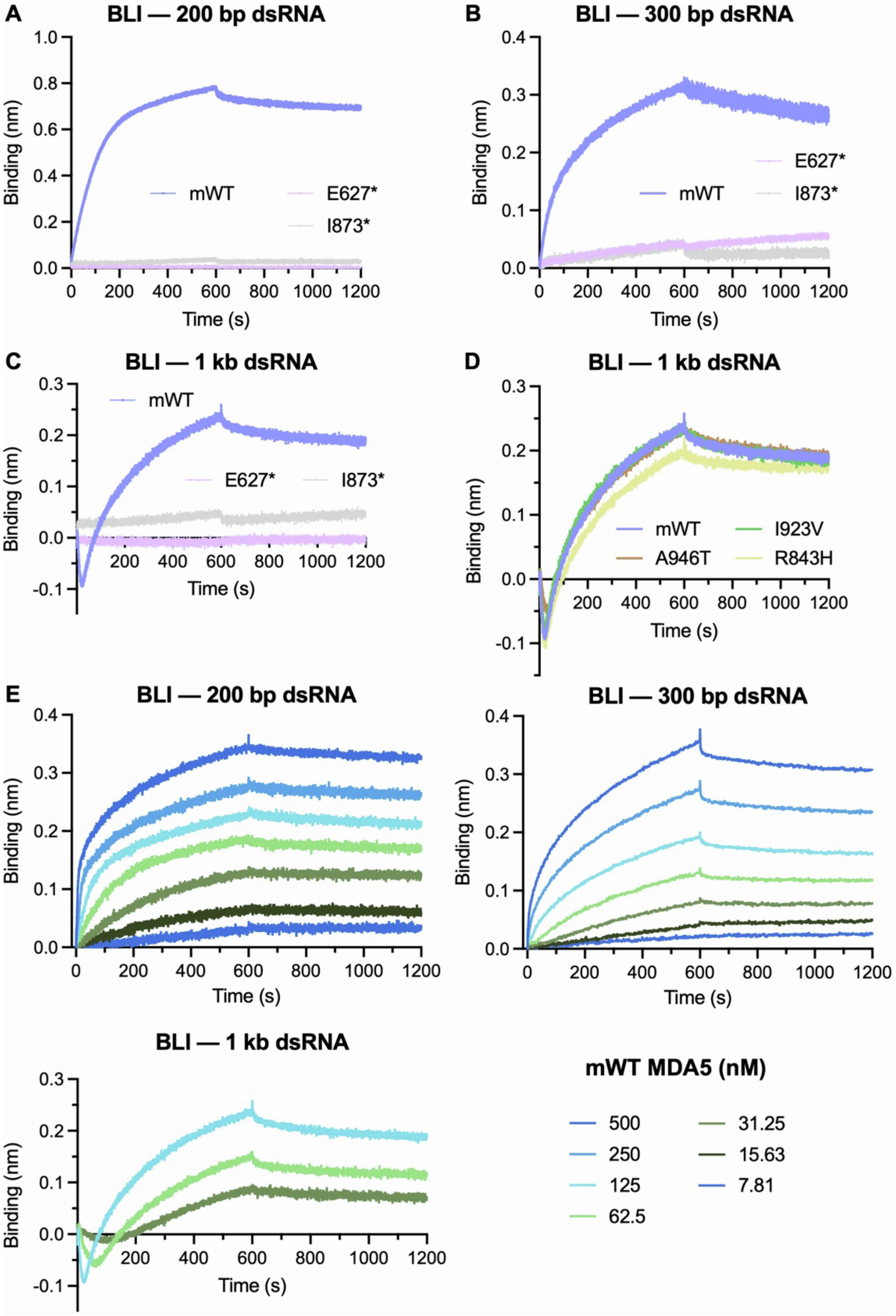

Fig. S2. Bio-layer interferometry (BLI). 3'-biotinylated dsRNA was immobilized on a streptavidin sensor and MDA5 was added to the mobile phase. (**A-C**) mouse wild type (mWT) MDA5 and truncation variants. (**D**) mWT and single-substitution MDA5 variants with 1-kb dsRNA. (**E**) BLI with different concentrations of mWT MDA5 and dsRNA ligands. The curves for 1-kb dsRNA in (*C-E*) have complex multiphasic shapes. Related to Figure 2.

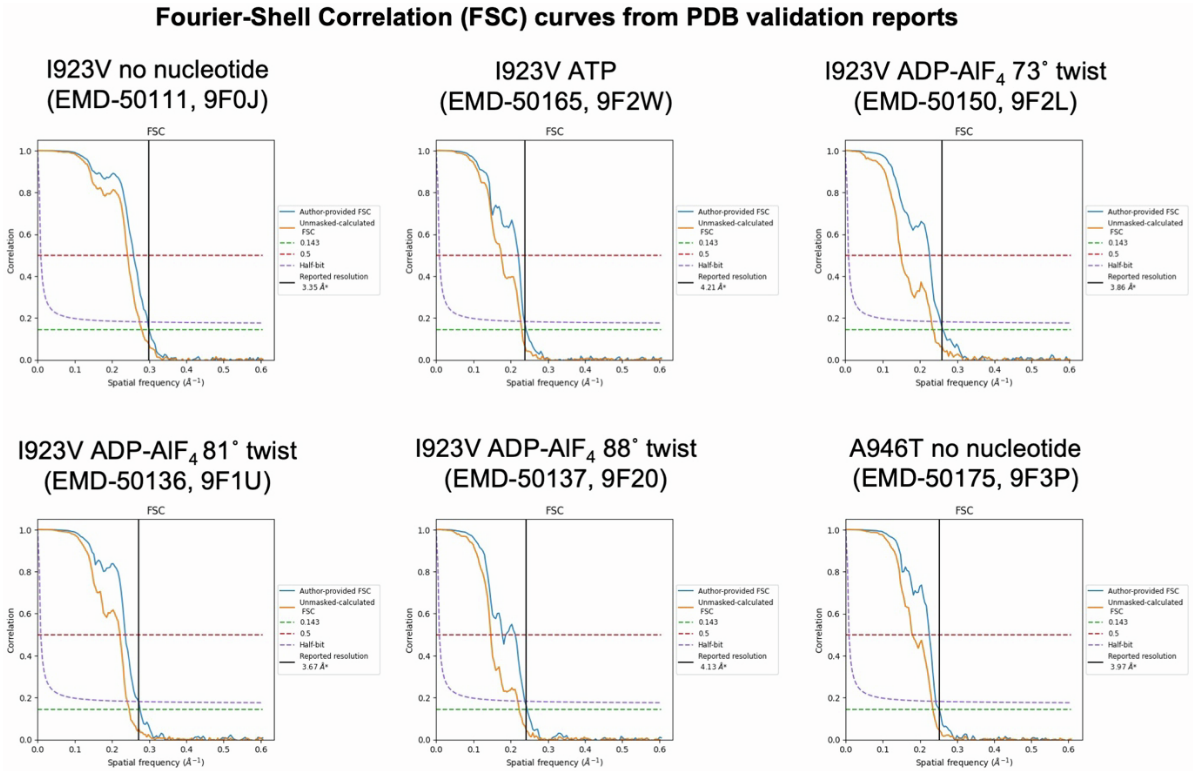

Fig. S3. Fourier shell correlation curves for cryo-EM image reconstructions. Electron Microscopy Data Bank (EMDB) and Protein Data Bank (PDB) accession codes are listed for each structure. Graphs were generated as part of the PDB validation reports. Related to Figure 3.

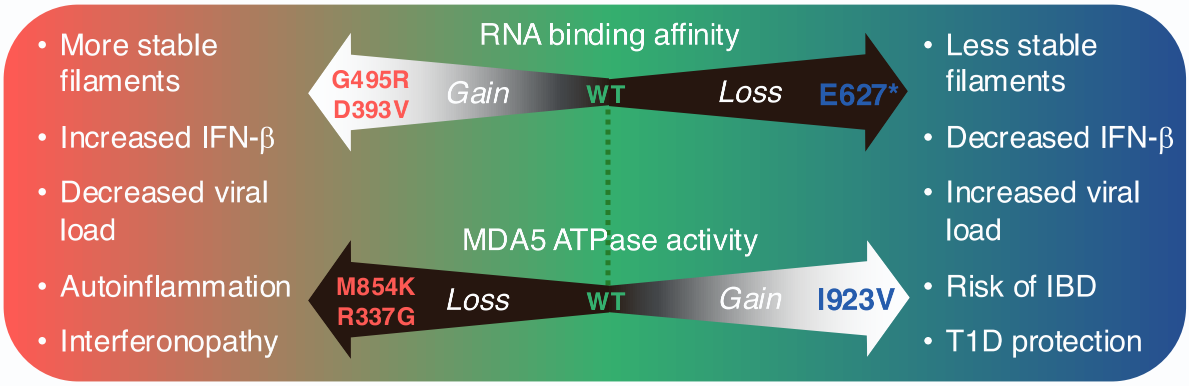

Fig. S4. MDA5 variants tune immune homeostasis via a fitness trade-off between viral clearance and tissue damage. T1D-protective MDA5 variants E627* and I923V lead to loss of signaling function. E627* causes loss of RNA binding whereas I923V hyperactivates ATPase activity, which promotes dissociation from RNA. Loss of signaling function is associated with increased viral loads and IBD onset, while reducing autoimmune tissue damage. Variants that increase RNA binding or inhibit ATPase activity, leading to a gain of signaling function and autoinflammation, but infections are cleared more effectively. Related to Figures 1-4.

Table S1. Cryo-EM data collection and structure determination parameters.

	**I923V No nt.**	**I923V ATP**		**I923V ADP-AlF$_4$**		**A946T No nt.**
**Data Collection and Processing**						
Microscope/Detector	Krios/K3	Krios/K3		Krios/K3		Krios/K3
Micrograph fluence (e$^-$ Å^{-2})	40 – 48	40 – 48		40 – 48		40 – 48
Exposure per frame (e$^-$ Å^{-2})	1.0	1.0		1.0		1.0
Nominal defocus range (µm)	-0.5 – -2.5	-0.5 – -2.5		-0.5 – -2.5		-0.5 – -2.5
Pixel size (Å)	0.826	0.826		0.826		0.822
N. initial segment images	1,008,651	683,122		1,406,812		1,378,603
**Map averaging and refinement**			**73° twist**	**81° twist**	**88° twist**	
N. final segment images	547,562	337,327	53,484	347,343	98,676	234,719
Resolution, unmasked ½-maps (Å)	3.57	4.35	4.25	4.05	4.48	4.25
Final resolution with masking (Å)	3.33	4.21	3.86	3.67	4.13	3.97
Max. local resolution range (Å)	5.94-3.12	7.31-3.80	7.91-3.48	6.58-3.35	7.85-3.84	6.52-3.67
Map sharpening B factor (Å^2)	-90	-130	-50	-85	-90	-150
Helical twist ($^\circ$)	91.63	84.15	73.94	80.71	88.20	89.44
Helical rise (Å)	44.84	43.92	42.78	43.86	44.50	44.58
**Atomic model fit in data**						
CC (mask), Phenix v1.21	0.81	0.74	0.78	0.73	0.74	0.76
CC (volume), Phenix v1.21	0.80	0.74	0.78	0.73	0.73	0.75
**Atomic model composition**						
N. non-hydrogen atoms	5782	5,981	6,129	5,979	6,128	6,067
Protein residues	640	675	686	673	681	682
RNA nucleotides	30	28	28	28	30	30
Ligand	None	ATP	ADP-AlF$_4$	ADP-AlF$_4$	ADP-AlF$_4$	None
Zn^{2+} ions	1	1	1	1	1	1
**Atomic model geometry, ADPs**						
Bond lengths (Å)	0.004	0.003	0.003	0.004	0.003	0.003
Bond angles ($^\circ$)	0.525	0.566	0.578	0.606	0.656	0.548
Protein min/max/mean ADP	26/150/94	76/196/140	82/163/117	80/201/147	90/218/160	41/163/106
Nucleotide min/max/mean ADP	17/72/41	57/90/71	67/79/72	63/106/83	88/119/100	32/62/41
Ligand min/max/mean ADP	None	136/224/138	122/188/126	147/221/151	151/215/157	None
**Validation**						
MolProbity score, Phenix v1.21	1.82	2.00	1.70	2.03	2.11	2.06
Clash score, Phenix v1.21	8.53	11.78	6.71	13.30	14.96	11.67
Rotamer outliers (%)	0.00	0.00	0.00	0.00	0.00	0.00
Ramachandran plot						
% favored	94.9	93.8	95.3	94.1	93.5	92.3
% allowed	5.1	6.2	4.7	5.9	6.5	7.7
% outliers	0.0	0	0.0	0.0	0.0	0.0
PDB code	9F0J	9F2W	9F2L	9F1U	9F20	9F3P
EMDB code	EMD-50111	EMD-50165	EMD-50150	EMD-50136	EMD-50137	EMD-50175

*ADPs, atomic displacement parameters
